# DYRK1A-mediated Cyclin D1 Degradation in Neural Stem Cells Contributes to the Neurogenic Cortical Defects in Down Syndrome

**DOI:** 10.1016/j.ebiom.2015.01.010

**Published:** 2015-01-17

**Authors:** Sònia Najas, Juan Arranz, Pamela A. Lochhead, Anne L. Ashford, David Oxley, Jean M. Delabar, Simon J. Cook, María José Barallobre, Maria L. Arbonés

**Affiliations:** a Department of Developmental Biology, Instituto de Biología Molecular de Barcelona, CSIC, and Centro de Investigación Biomédica en Red de Enfermedades Raras (CIBERER), 08028 Barcelona, Spain; b Signalling Programme, The Babraham Institute, Babraham Research Campus, CB22 3AT Cambridge, UK; c Proteomics Group, The Babraham Institute, Babraham Research Campus, CB22 3AT Cambridge, UK; d Sorbonne Universités, UPMC Univ Paris 06, Inserm, CNRS, UM 75, U 1127, UMR 7225, ICM, 75013 Paris, France

**Keywords:** DS, Down syndrome, IP, intermediate progenitor, NSC, neural stem cell, RG, radial glia, SVZ, subventricular zone, VZ, ventricular zone, Cell cycle regulation, DYRK kinases, Cerebral cortex development, Trisomy 21, Neurodevelopmental disorders, Intellectual disability

## Abstract

Alterations in cerebral cortex connectivity lead to intellectual disability and in Down syndrome, this is associated with a deficit in cortical neurons that arises during prenatal development. However, the pathogenic mechanisms that cause this deficit have not yet been defined. Here we show that the human DYRK1A kinase on chromosome 21 tightly regulates the nuclear levels of Cyclin D1 in embryonic cortical stem (radial glia) cells, and that a modest increase in DYRK1A protein in transgenic embryos lengthens the G1 phase in these progenitors. These alterations promote asymmetric proliferative divisions at the expense of neurogenic divisions, producing a deficit in cortical projection neurons that persists in postnatal stages. Moreover, radial glial progenitors in the Ts65Dn mouse model of Down syndrome have less Cyclin D1, and *Dyrk1a* is the triplicated gene that causes both early cortical neurogenic defects and decreased nuclear Cyclin D1 levels in this model. These data provide insights into the mechanisms that couple cell cycle regulation and neuron production in cortical neural stem cells, emphasizing that the deleterious effect of *DYRK1A* triplication in the formation of the cerebral cortex begins at the onset of neurogenesis, which is relevant to the search for early therapeutic interventions in Down syndrome.

## Highlights

• Kinase DYRK1A regulates Cyclin D1 levels and cell cycle duration in progenitors of the developing cerebral cortex.
• *DYRK1A* gene-dosage imbalance alters the neurogenic output of cortical progenitors at the onset of neurogenesis.
• Normalization of *Dyrk1a* gene-dosage restores early cortical neurogenesis in a trisomic mouse model for Down syndrome.

## 1 Introduction

The mammalian neocortex is the brain region responsible for cognitive function, sensory perception and consciousness. It is formed by many types of neurons and glial cells, all of which are distributed across six histologically defined layers that are generated in a spatially and temporally-regulated manner thanks to the interplay of intrinsic molecular programs and extracellular cues (Tiberi et al., 2012). Impaired development of this brain structure has been associated with mental deficiency and other major neurological disorders (Lewis and Sweet, 2009, Rubenstein, 2010, Sun and Hevner, 2014).

Around 80% of neocortical neurons are excitatory projection neurons that extend axons to distant intracortical targets and to subcortical regions, whilst the remainder are inhibitory interneurons involved in local circuits (DeFelipe et al., 2013, Greig et al., 2013). The distinct types of projection neurons are produced in the dorsolateral telencephalon (*pallium*) of the embryo from multipotent neural stem cells (NSCs) known as radial glia (RG), and from more restricted progenitors, the intermediate progenitors (IPs). These neurons are generated in an inside–outside pattern, first generating the neurons that form the layer closest to the ventricle (layer VI) and lastly those that form the most superficial layers (Layers II–III). Projection neurons within a layer have common molecular characteristics and connectivity patterns, which they acquire at their birth (Greig et al., 2013, Molyneaux et al., 2007).

During neurogenesis, RG progenitors divide asymmetrically in the ventricular zone (VZ) producing another RG cell and either a neuron or an IP (Noctor et al., 2004, Haubensak et al., 2004, Miyata et al., 2004). This progenitor moves to a more basal proliferative layer, the subventricular zone (SVZ), where it divides symmetrically to produce a pair of neurons directly or it does so after 1 to 3 rounds of symmetric amplifying divisions (Noctor et al., 2004, Kowalczyk et al., 2009). Consequently, as neocortical development progresses and the cellularity in the SVZ increases, IPs become the major source of projection neurons (Breunig et al., 2011, Kowalczyk et al., 2009). According to this model, the number and proportion of projection neuron subtypes in a cortical radial column are related to the number of RG progenitors that are present at the onset of neurogenesis, as well as to the number of VZ (apical) and SVZ (basal) proliferative and neurogenic divisions (Huttner and Kosodo, 2005, Noctor et al., 2001, Noctor et al., 2007).

Regulation of the cell cycle, and particularly of the G1 phase of the cell cycle, is important for the normal expansion of the neocortex in both rodents and primates (Dehay and Kennedy, 2007). G1 is a critical phase, integrating extracellular signals that induce either commitment to a further round of cell division, or withdrawal from the cell cycle and differentiation (Cunningham and Roussel, 2001, Dehay and Kennedy, 2007, Salomoni and Calegari, 2010, Zetterberg et al., 1995). Pioneering cumulative S-phase labelling experiments performed in the mouse embryo showed that as neurogenesis progresses the cell cycle of neocortical progenitors extends due to a progressive lengthening of the G1 phase (Takahashi et al., 1995). Moreover, there is evidence of a correlation between cell cycle length and neurogenesis, which has led to the formulation of the cell cycle length hypothesis (Gotz and Huttner, 2005). According to this hypothesis, the time that a progenitor spends in G1 determines the final effect of a particular cell fate determinant, which could be equivalent (symmetric divisions) or distinct (asymmetric divisions) in the two daughter cells (Dehay and Kennedy, 2007, Gotz and Huttner, 2005, Salomoni and Calegari, 2010). Indeed, it was more recently shown that manipulating the duration of the G1 phase in neocortical apical progenitors alters the production of IPs and neurons (Lange et al., 2009, Pilaz et al., 2009).

Down syndrome (DS), the most common genetic cause of intellectual disability, is caused by trisomy of chromosome 21. DS brains are smaller than normal brains and they exhibit neuronal deficits in several regions, including the cerebral cortex (Ross et al., 1984). Infants with DS also present hypocellularity in this brain structure (Schmidt-Sidor et al., 1990, Wisniewski, 1990), indicating that defects in prenatal development are a major determinant of the deficit in adults. Indeed, fewer cells (Larsen et al., 2008) and disorganized laminas are evident in the cerebral cortex of DS foetuses from as early as the second trimester of gestation (Golden and Hyman, 1994).

The availability of DS mouse models in which different regions of chromosome 21 are in trisomy (Haydar and Reeves, 2012, Liu et al., 2011) has allowed the effect of trisomic genes on prenatal development to be assessed, assigning phenotypic aspects of the syndrome to a region of chromosome 21. In the best studied model of DS, the Ts65Dn mouse (Reeves et al., 1995), the growth of the neocortical wall is delayed due to the impaired production of neurons early in neurogenesis that is concomitant with a lengthening of the cell cycle in the ventricular germinal layer (Chakrabarti et al., 2007). The Ts1Cje mouse is a DS model with a smaller trisomic region than the Ts65Dn mouse (Haydar and Reeves, 2012), yet it also develops an abnormally thin neocortex and slower cell cycle exit is observed during embryogenesis (Ishihara et al., 2010). Importantly, the proliferation markers expressed in the neocortical germinal matrix of DS foetuses also suggest cell cycle defects which underpin the reduced neuron production (Contestabile et al., 2007). There are around 80 genes in the triplicated segment common to Ts65Dn and Ts1Cje mice, which contains the DS critical region (*DSCR*) of chromosome 21 (Delabar et al., 1993, Toyoda et al., 2002). Thus, it is likely that dosage imbalance of one or a few genes in this region contributes to the deficit of cortical neurons in DS.

In this study we have assessed the possibility that triplication of *DYRK1A*, a *DSCR* gene, contributes to the hypocellularity of the cerebral cortex associated with DS. DYRK1A (dual-specificity tyrosine-(Y)-phosphorylation regulated kinase 1A) encodes a constitutively active kinase that phosphorylates serine and threonine residues in a variety of substrates (Becker and Sippl, 2011). In humans, truncating mutations in the *DYRK1A* gene cause primary microcephaly (Courcet et al., 2012) and autism (O'Roak et al., 2012). Moreover, mice and flies with haploinsufficiency of the *Dyrk1a*/*minibrain* genes have smaller brains (Fotaki et al., 2002, Tejedor et al., 1995), indicating that the role of DYRK1A in brain growth is conserved across evolution. Experiments on neural progenitors derived from induced pluripotent stem cells from monozygotic twins discordant for trisomy 21 highlight *DYRK1A* as one of the chromosome 21 genes important for the proliferation and differentiation defects associated with DS (Hibaoui et al., 2014). However, despite the evidence from different model systems showing that DYRK1A regulates neural proliferation and differentiation (Tejedor and Hammerle, 2011), the pathogenic effects of DYRK1A overexpression in the formation of brain circuits in DS remain unclear (Haydar and Reeves, 2012).

The effect of DYRK1A overexpression on cortical neurogenesis has been assessed in the mouse embryo through electroporation, although the results obtained were inconclusive. The ectopic overexpression of DYRK1A in progenitors of the dorsal telencephalon induced proliferation arrest (Hammerle et al., 2011, Yabut et al., 2010), provoking premature neuronal differentiation (Yabut et al., 2010), a phenotype that is quite opposite to the growth delay of the cortical wall observed in the Ts65Dn embryos (Chakrabarti et al., 2007). These studies involved electroporation at mid-corticogenesis stages and the levels of DYRK1A overexpression were not controlled. More recent experiments showed that modest DYRK1A overexpression does not disturb the birth of cortical neurons when induced at the onset of neurogenesis (Kurabayashi and Sanada, 2013). Thus, the effect of DYRK1A on cortical neurogenesis seems to depend on the time and/or the level of overexpression.

Using mouse models that overexpress *Dyrk1a* under its endogenous regulatory sequences, mimicking the situation in DS, we now demonstrate that trisomy of *Dyrk1a* is sufficient to lengthen the G1 phase of the cell cycle and to bias the production of RG-derived neurons and IPs during the early phase of corticogenesis, and that the triplication of the *Dyrk1a* gene is necessary for dampened early neurogenesis in the developing neocortex of Ts65Dn embryos.

## 2 Materials and Methods

### 2.1 Animals

In this study we have used embryos and postnatal *Dyrk1a*^+/−^ mice, mBACTg*Dyrk1a* mice, Ts65Dn mice and their respective wild-type littermates, as well as the mice resulting from crosses between Ts65Dn females and *Dyrk1a*^+/−^ males. The day of the vaginal plug was defined as E0.5, and the day of birth was defined as P0.

The generation of Ts65Dn mice, *Dyrk1a*^+/−^ mice and mBACtg*Dyrk1a* mice was described elsewhere (Davisson et al., 1993, Fotaki et al., 2002, Guedj et al., 2012). Mice were maintained in their original genetic backgrounds: *Dyrk1a*^+/−^ mice by repeated backcrossing of *Dyrk1a*^+/−^ males to C57BL/6Jx129S2/SvHsd F1 females (Harlan Laboratories); mBACtg*Dyrk1a* mice by repeated backcrossing of transgenic males to C57BL6/J females (Charles River Laboratories); and Ts65Dn mice by repeated backcrossing of parental Ts65Dn females (Jackson Laboratory, USA) to B6EiC3 males (Harlan laboratories). *Dyrk1a*^+/−^ and mBACtg*Dyrk1a* mice were genotyped by PCR (Fotaki et al., 2002, Guedj et al., 2012) and Ts65Dn mice by PCR (Reinholdt et al., 2011) or by quantitative PCR (http://www.jax.org/cyto/quanpcr.html).

All the experimental procedures were carried out in accordance with the European Union guidelines (Directive 2010/63/EU) and the followed protocols were approved by the ethics committee of the Parc Científic de Barcelona (PCB).

### 2.2 Tissue Preparation for Histology

To obtain embryonic tissue, whole heads were fixed by immersion in 4% paraformaldehyde (PFA) for 24 h at 4 °C, cryoprotected with 30% sucrose in PBS, embedded in Tissue-Tek O.C.T. (Sakura Finetek), frozen in isopentane at − 30 °C and sectioned on a cryostat. Cryosections (14 μm) were collected on Starfrost precoated slides (Knittel Glasser) and distributed serially. Postnatal P0 and P7 mice were deeply anaesthetized in a CO_2_ chamber and transcardially perfused with 4% PFA. The brains were removed, post-fixed and cryoprotected as indicated above, and cryotome (40 μm) sections were then distributed serially. For DYRK1A and PDGFRα immunostainings in embryos, the post-fixed brains were embedded in 2% agarose and sectioned directly on a vibratome (40 μm).

### 2.3 Immunostainings and Cell Counts

For accurate immunostaining with some antibodies (see Supplementary information) it was necessary to perform an antigen retrieval treatment before blocking: sections were boiled for 10 min in sodium citrate buffer (2 mM citric acid monohydrate, 8 mM tri-sodium citrate dihydrate, pH 6.0). For BrdU immunostaining, sections were incubated before blocking in 50% formamide in 2 × SSC at 64 °C for 10 min followed by an incubation in 2 N HCL at 37 °C for 30 min and finally 10 min in 0.1 M boric acid (pH 8.5) at room temperature (RT). The sections were blocked for 1 h at RT in PBS containing 0.2% Triton-X100 and 10% foetal bovine serum (FBS) and probed for 12 to 48 h at 4 °C with the primary antibodies diluted in antibody buffer (AB: PBS containing 0.2% Triton-X100 and 5% FBS). Sections were washed and primary antibodies detected by using Alexa-555 and Alexa-488 conjugated secondary antibodies (1:1.000; Life Technologies). Cell nuclei were stained with Hoechst (Sigma-Aldrich). For antibodies against BrdU and Pax6, and against Cyclin D1 (in E13.5, E16.5 and E18.5 brain sections) signal amplification was required: after washing primary antibodies, sections were incubated with the corresponding biotinylated secondary antibody (1:200; Vector Labs), washed and incubated with Alexa-488 conjugated streptavidin (Life Technologies). In all cases, the specificity of the immunoreaction was tested by omitting the primary antibody. A complete list of the primary antibodies can be found in Supplementary data.

Images were taken using the Leica TCS SP5 confocal microscope or the Leica AF7000 motorized wide-field microscope. Labelled cells in embryonic sections were counted in a 100 μm-wide column of the lateral cortical wall, with the exception of cleaved-caspase3^+^ cells that were counted in a 400 μm-wide column at E11.5 and in 1400 μm width column at E14.5, and pH3^+^ cells, Olig2^+^ cells and EdU-labelled cells that were counted in 600 μm-, 300 μm- and 250 μm-wide columns, respectively. Labelled cells in postnatal sections were counted in a wide column of 350 μm in Fig. 6B and in a wide column of 200 μm in Fig. 7D and Supplementary Figs. 9 and 12. All cell counts were blind and performed in a minimum of 3 sections of the same rostro-caudal level per embryo or mice.

### 2.4 Measurements of Cytoplasmic and Nuclear Cyclin D1

Relative cytoplasmatic and nuclear Cyclin D1 protein levels were estimated in confocal images of brain sections stained for Cyclin D1 and the nuclei labelled with Hoechst. First, images were converted to binary images by applying a threshold level on them using the Image-J software. Cytoplasmatic Cyclin D1 was assigned to the Cyclin D1 signal that did not overlap with the Hoechst signal and was obtained by subtracting the Hoechst binary image to the Cyclin D1 binary image. Nuclear Cyclin D1 corresponded to the Cyclin D1 signal that overlapped with the Hoechst signal and was obtained by subtracting the cytoplasmatic Cyclin D1 binary image to the total Cyclin D1 binary image (see Supplementary Fig. 2C and D). Labelling intensities of total, nuclear and cytoplasmatic Cyclin D1 were obtained in each image using the integrated density option of the Image-J software. Cyclin D1 measurements were done in the medial region of the dorso-lateral VZ within a rectangle of 250 μm × 50 μm in E11.5 and E13.5 sections or in a rectangle of 250 μm × 40 μm in E16.5 sections. As a consequence of the interkinetic nuclear movement of RG progenitors (Gotz and Huttner, 2005), the majority of progenitors with their nuclei in the intermediate VZ region should be in G1 or in G2.

### 2.5 Cell Cycle Exit Rates and Cell Cycle Parameters

For cell cycle exit rate quantifications, pregnant females were intraperitoneally injected with one pulse of BrdU (100 mg/kg; Sigma) and sacrificed 24 h later. Embryos were collected and processed as describe above. Sections were immunostained for BrdU and Tbr1 (E12.5 embryos) or for BrdU and Ki67 (E14.5 embryos). Neuronal production was estimated in E12.5 embryo sections counting the proportion of BrdU immunolabelled cells that were Tbr1^+^. Cell cycle exit rates were estimated in E14.5 embryo sections counting the proportion of BrdU immunolabelled cells that were negative for Ki67.

Cell cycle duration of radial glial progenitors was measured *in vivo* following the S-phase cumulative EdU labelling protocol described by Arai et al. (2011). Briefly, pregnant females (E11.5) were repeatedly injected with EdU (3.3 mg/kg; Life Technologies), every hour for the first five injections and every 2 h for the remaining ones, and sacrificed at different time points according to the schedule shown in Fig. 2A. Embryos were post-fixed, cryoprotected and cryosectioned as described before. EdU was detected in coronal sections using the Click-iT EdU Alexa Fluor 647 kit (Life Technologies) according to manufacturer's instructions. EdU cell counts were performed in a 250 μm-wide field of the dorsolateral wall in a minimum of 3 sections per embryo. The proportion of Hoechst-stained nuclei in the VZ that were EdU^+^, Tuj1^−^ and Tbr2^−^ at the different times of cumulative labelling (labelling index) were plotted (see Fig. 2C) as described previously (Nowakowski et al., 1989, Takahashi et al., 1995) to estimate the growth fraction, GF (fraction of cells that are proliferating and that correspond to the maximum labelling index); the cell cycle duration, Tc; and the S-phase duration, Ts. Tc and Ts were calculated taking into account the Tc–Ts value, which is the cumulative labelling time required to achieve the maximum labelling index, and the Ts/Tc value was given by the intersection of the extrapolation of the linear regression line to the Y axis. To generate the best fitted-regression lines we applied the Linear Regression Model of the Prism software (version 5; GraphPad software) to the averaged labelling indexes of the time points before reaching the GF. The slopes of the best-fitted lines in wild-type and Tg*Dyrk1a* samples were analysed for statistical significance with the same software.

For estimating duration of G2 and M phases, sections from embryos exposed to EdU for 1, 2 or 3 h were immunostained against pH3 to identify mitotic progenitors and EdU visualized as before. Mitotic progenitors at the apical surface were counted in a 250 μm-wide field in a minimum of 3 sections per embryo. G2 duration was considered as the time required for half-maximal appearance of EdU in mitotic progenitors (see Fig. 2D), and M phase duration to the proportion of VZ progenitors that were in mitosis (pH3^+^ cells) multiply to the total cell cycle duration as described in Arai et al. (2011). All these values were then used to calculate the duration of G1 phase.

### 2.6 Western Blotting

Total protein extracts (≈ 40 μg) were resolved by SDS-PAGE and transferred onto a nitrocellulose membrane (Hybond-ECL, Amersham Biosciences) or Immobilon P membranes (Millipore) that was probed with antibodies whose binding was detected by infrared fluorescence using the LI-COR Odyssey IR Imaging System V3.0 (LI-COR Biosciences) or by chemiluminescence using the Amersham ECL™ Western blotting detection reagent (Amersham Life Sciences) and X-ray Films (AGFA). Primary antibodies used were: mouse monoclonal anti-DYRK1A (1:500; Abnova Corporation or 1:1000; Santa Cruz), anti-p27 (1:500; BD Biosciences), anti-p21 (1:200; Santa Cruz), anti-vinculin (1:5000; Sigma-Aldrich) and anti-Cyclin D1 (1:200, Calbiochem); rabbit polyclonal anti-Cyclin D1 (1:2,000; Thermo Scientifics), anti-actin (1:5000; Sigma-Aldrich), anti-retinoblastoma (1:500; BD Biosciences), anti-HSP90 (1:2000; BD Biosciences), anti-pT286-cyclin D1 (1:500; Cell Signalling), anti-GFP (1:1000, Roche) and anti-RCAN1 (1:1000). Polyclonal HA antibody conjugated to agarose beads was from Santa Cruz. Secondary antibodies for infrared fluorescent detection were goat anti-mouse IgG IRDye-800CW and goat anti-rabbit IgG IRDye-680CW, and for chemiluminescence detection were rabbit anti-mouse and goat anti-rabbit IgG conjugated to horseradish peroxidase (1:2000; Dako).

### 2.7 RNA Extraction and Real-time qPCR

Total RNA from the telencephalon of E10.5 and E11.5 embryos were extracted using the RNeasy Mini kit (Qiagen) according to manufacturer's instructions and the eluted RNA treated with DNAse (Ambion) for 30 min at 37 °C. cDNAs were synthesized from 1 μg of total RNA using Superscript II retrotranscriptase (Life Technologies) and random hexamers (Life Technologies). Real-time qPCR was carried out with the Lightcycler 480 platform (Roche) using SYBR Green I Master Kit (Roche). *Peptidyl-prolyl isomerase A* (*Ppia*) was used as reference gene for data normalization. A complete list of the primers used can be found in Supplementary data.

### 2.8 Statistical Analysis

Data are presented as the mean ± S.E.M. and were analysed by the two-tailed Student's t-test with the exception of the EdU labelling index data that was analysed as indicated before with the Linear Regression Model of the Prism software. A minimum of three embryos or mice of the same genotype was analysed in each experiment. Differences were considered significant at p-values < 0.05: *p < 0.05, **p < 0.001 and ***p < 0.0001.

## 3 Results

### 3.1 Neocortical RG Progenitors in Tg*Dyrk1a* Embryos Have Reduced Levels of Nuclear Cyclin D1

**Fig. 1:**
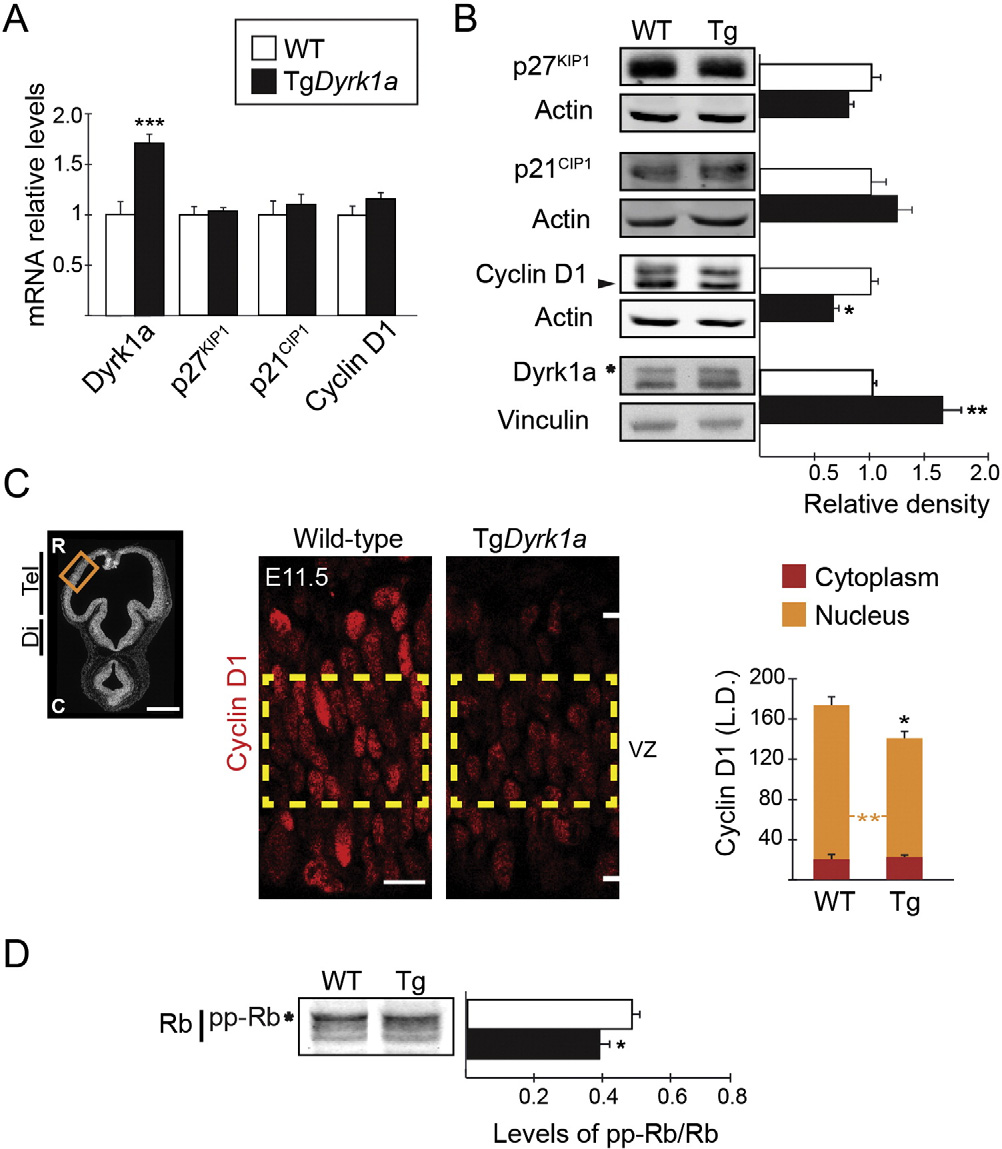
Tg*Dyrk1a* RG progenitors show decreased levels of nuclear Cyclin D1 and hyperphosphorylated retinoblastoma. (A) Relative mRNA levels of *Dyrk1a*, *p27^KIP1^*, *p21^CIP1^* and *Cyclin D1* determined by RT-PCR on mRNA obtained from the telencephalon of E11.5 wild-type (WT) and Tg*Dyrk1a* embryos. (B) Representative Western blots of extracts prepared from the telencephalon of E11.5 embryos and probed with the indicated antibodies. Histograms show the protein levels in Tg*Dyrk1a* embryos normalized to actin or vinculin levels and expressed relative to the WTs. Arrowhead indicates the band corresponding to the Cyclin D1 isoform that contains T286 and asterisk indicates the band corresponding to Dyrk1a. (C) Picture of an E11.5 coronal brain section with nuclei visualized with Hoechst indicating the region in the dorsal telencephalon (Tel) where quantifications were done (image on the left), and WT and Tg*Dyrk1a* (Tg) sections immunostained for Cyclin D1 (images on the right). The histogram shows the labelling densities (L.D.) of Cyclin D1 fluorescence signals in the nucleus and cytoplasm of radial glial progenitors of the ventricular zone (VZ) region indicated by the yellow square and calculated as shown in Supplementary Fig. 2C and D. C, caudal; Di, Diencephalon; Tel, Telencephalon; R, rostral. (D) Representative Western blot and its quantification showing the levels of retinoblastoma (pRb) that is hyperphsophorylated (pp-Rb) in E11.5 WT and Tg*Dyrk1a* total telencephalic extracts. Histogram values are the mean ± S.E.M. *P < 0.05, ***P < 0.001 (n ≥ 3). Bars = 500 μm (left picture in C) and 20 μm (right pictures in C).

During the neurogenic phase of neocortical development, DYRK1A is expressed in progenitors of the VZ (Pax6^+^ cells) as well as in progenitors of the SVZ (Tbr2^+^ cells) (Supplementary Fig. 1). As ectopic overexpression of DYRK1A induces cell cycle exit in neural cells (Park et al., 2010, Yabut et al., 2010), we asked whether triplication of *Dyrk1a* gene is sufficient to affect the expression of cell cycle regulators in cortical neural progenitors *in vivo*. To this end, we used a transgenic mouse model, the mBACtg*Dyrk1a* mouse (Tg*Dyrk1a* hereafter), which carries in a BAC the whole *Dyrk1a* gene (Guedj et al., 2012). There is evidence showing that DYRK1A can induce cell cycle exit in neural progenitors by different means; promoting the nuclear export and degradation of the cell cycle activator Cyclin D1 (Yabut et al., 2010), inducing the expression of the Cdk inhibitors *p27^KIP1^* (Hammerle et al., 2011) and *p21^CIP1^* (Park et al., 2010), and promoting the stabilization of p27^KIP1^ protein (Soppa et al., 2014). Therefore, we compared the mRNA and protein levels of these cell cycle regulators in the telencephalon of wild-type and Tg*Dyrk1a* embryos at the beginning of neurogenesis (E11.5), which is mainly formed by the germinal VZ (see Supplementary Fig. 1A). Consistent with the genetic complement, Dyrk1a mRNA and protein levels were increased (1.5–1.7 fold) in the telencephalon of transgenic embryos when compared to the wild-type (Fig. 1A and B). mRNA and protein levels of the cell cycle regulators examined were the same in both genotypes with the exception of Cyclin D1 protein levels that were decreased in the Tg*Dyrk1a* embryos (Fig. 1A and B). Similarly, E10.5 Tg*Dyrk1a* whole embryos exhibited reduced Cyclin D1 protein levels but normal levels of Cyclin D1, *p27^KIP1^* and *p21^CIP1^* mRNA transcripts (Supplementary Fig. 2A and B). Thus, a 1.5-fold increase in DYRK1A at the beginning of neurogenesis diminished Cyclin D1 protein content in the mouse embryo irrespective of the cell type. We also performed immunostaining for Cyclin D1 and found that the levels of Cyclin D1 in the nuclei of RG progenitors were significantly decreased in E11.5 Tg*Dyrk1a* embryos (Fig. 1C and Supplementary Fig. 2C and D).

One of the main nuclear functions of Cyclin D1 is to promote G1-to-S phase transition through association with and activation of Cdk4/6. This leads to the phosphorylation of the retinoblastoma protein (pRb), which promotes the release of E2F transcription factor from the pRb/E2F complex and the expression of genes necessary for cell cycle progression (Cunningham and Roussel, 2001). Consistent with the decreased levels of nuclear Cyclin D1 (Fig. 1C), the relative amount of hyperphosphorylated Rb in the telencephalon of E11.5 Tg*Dyrk1a* embryos was lower than in the wild-types (Fig. 1D), indicating that a 1.5-fold increase in DYRK1A is sufficient to reduce Cyclin D-Cdk activity in telencephalic RG progenitors at the onset of neurogenesis.

Nuclear export of Cyclin D1 and its subsequent degradation *via* the ubiquitin–proteasome pathway requires the phosphorylation of Cyclin D1 at threonine 286 (T286) (Diehl et al., 1997). Whilst glycogen synthase kinase 3 beta (GSK3β) was thought to be the only kinase responsible for phosphorylating Cyclin D1 on T286 (Diehl et al., 1998), the demonstration that DYRK1A could promote the nuclear export and turnover of Cyclin D1 in Neuro2a progenitor cells (Yabut et al., 2010) raised the possibility that DYRK1A might also be a ‘T286 kinase’, providing a mechanism for the reduction in Cyclin D1 levels. However, since the class I DYRKs (DYRK1A and DYRK1B) can also act as priming kinases for GSK3β, attribution of DYRK1A as a T286 kinase requires careful mapping of phosphorylation *in vitro* and *in vivo*. Indeed, we recently showed unambiguously that DYRK1B was able to phosphorylate Cyclin D1 directly at T286 *in vitro* and in cells independently of GSK3β (Ashford et al., 2014). Here we applied the same analysis to determine if DYRK1A could phosphorylate Cyclin D1 at T286. First, using [γ-^32^P] ATP in *in vitro* kinase reactions, we found that recombinant DYRK1A could phosphorylate purified recombinant wild-type Cyclin D1 but not Cyclin D1-T286A (Supplementary Fig. 3A). Second, recombinant wild-type Cyclin D1 that was phosphorylated by DYRK1A in these *in vitro* reactions was detected by a phospho-specific pT286 antibody whereas Cyclin D1-T286A was not (Supplementary Fig. 3B). These results demonstrated that DYRK1A could directly phosphorylate Cyclin D1 at T286 *in vitro*. To assess whether DYRK1A could also promote pT286 in cells we co-expressed DYRK1A and Cyclin D1 in HEK293 cells and mapped phosphorylation by mass spectrometry. As with DYRK1B previously (Ashford et al., 2014), we detected only a single phospho-peptide and this was phosphorylated at T286 (Supplementary Fig. 3C). Consistent with this, co-expression of DYRK1A and Cyclin D1 promoted phosphorylation of Cyclin D1 at T286 as detected by the pT286 antibody (Supplementary Fig. 3D). Finally, a catalytically inactive mutant of DYRK1A (KD, kinase dead, K188R) failed to promote pT286 and the pT286 antibody failed to detect DYRK1A-driven phosphorylation of Cyclin D1-T286A in cells (Supplementary Fig. 3E). These results confirm and extend published work (Chen et al., 2013, Soppa et al., 2014) and show that as with DYRK1B (Ashford et al., 2014) DYRK1A is a bona fide Cyclin D1 kinase that can phosphorylate T286.

The mouse *Cyclin D1* gene, like its human counterpart, expresses two spliced mRNA variants (Wu et al., 2009); one encodes the canonical Cyclin D1 isoform that contains T286 in its carboxy-terminus, and the other encodes a longer protein isoform with a distinct carboxy-terminal domain that lacks the phosphorylation consensus sequence for DYRK kinases. We could not directly assess Cyclin D1 phosphorylation in Tg*Dyrk1a* embryos because pT286 antibodies failed to detect endogenous phosphorylated Cyclin D1. However, our immunofluorescence and Western blot results showed reduced levels of nuclear Cyclin D1 and the specific loss of the shorter Cyclin D1 isoform bearing T286 (Fig. 1B–C and Supplementary Fig. 2B) in Tg*Dyrk1a* embryos, strongly suggesting that DYRK1A phosphorylates T286 in Cyclin D1 *in vivo* to regulate Cyclin D1 levels.

### 3.2 Tg*Dyrk1a* RG Progenitors of the Dorsal Telencephalon Have Longer Cell Cycle Duration

**Fig. 2:**
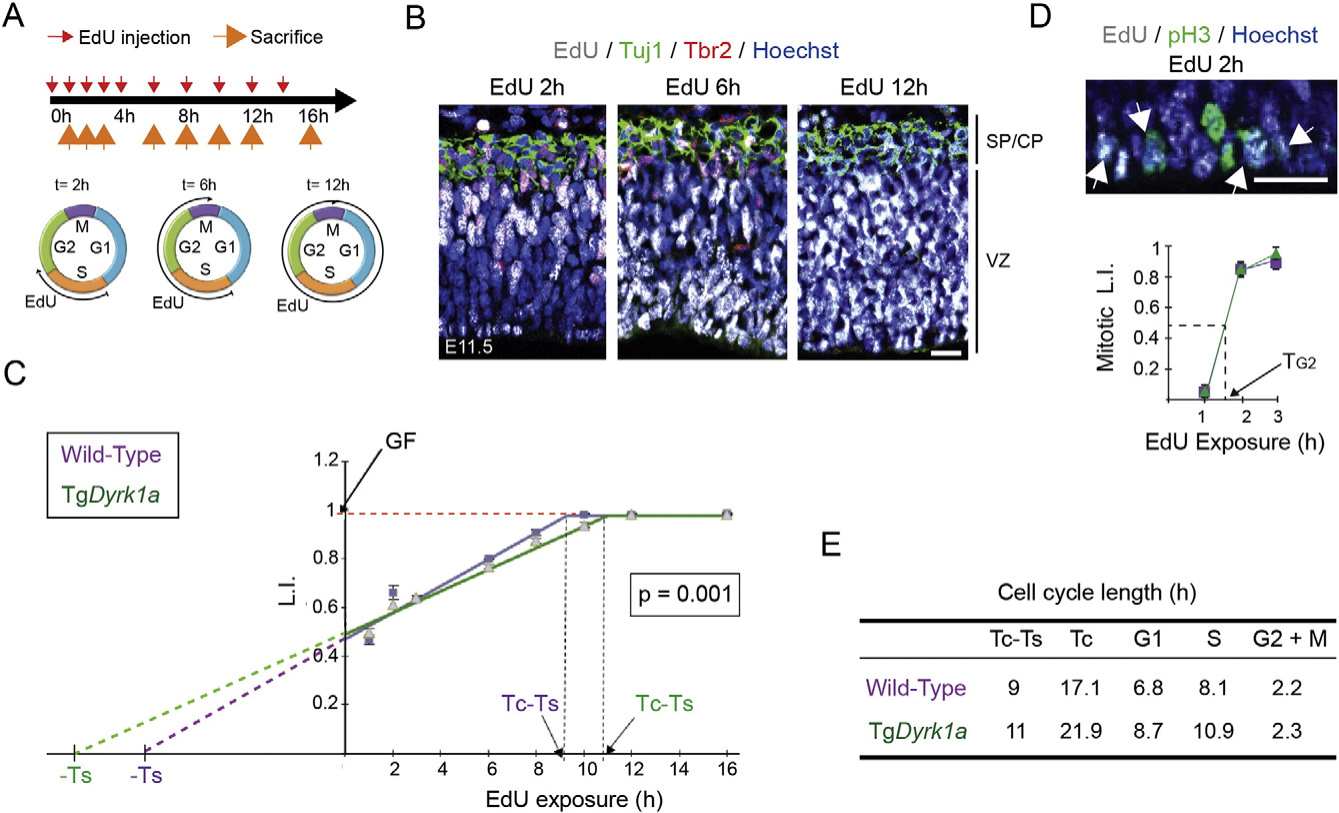
Tg*Dyrk1a* RG progenitors have longer cell cycles due to an enlargement of the G1 and S phases. (A) Schedule of the cumulative EdU-labelling protocol employed to calculate the cell cycle parameters in E11.5 radial glial progenitors of the dorsal telencephalon. Red and orange arrows indicate the time points at which EdU was injected and the time points at which embryos were harvested, respectively. (B) Representative confocal images of brain sections from wild-type embryos exposed to EdU for the indicated time and stained for EdU, Tuj1 and Tbr2. Nuclei were visualized with Hoechst. CP, cortical plate; SP, subplate; VZ, ventricular zone. (C) Plot showing the labelling indexes (L.I.; proportion of EdU-labelled nuclei) of radial glial progenitors (Tbr2^−^, Tuj1^−^ cells in the VZ) in wild-type and Tg*Dyrk1a* embryos at the indicated time of EdU exposure. GF, growth fraction (dashed red line); Tc, cell cycle duration; Ts, S-phase duration; Tc-Ts, time at which the labelling index reaches the GF (dashed black lines). (D) Representative image of a section from a wild-type embryo (2 h EdU exposure) stained for EdU and pH3 and nuclei visualized with Hoechst. The plot shows the mitotic L.I. (proportion of EdU-labelled radial glial progenitors in mitosis (pH3^+^)) at the indicated times of EdU exposure in wild-type and Tg*Dyrk1a* embryos. TG2, G2-phase duration. Arrows in the image point to the mitotic nuclei (green) that were labelled for EdU (grey). (E) Table summarizing the cycle parameters of wild-type and Tg*Dyrk1a* radial glial progenitors. Estimation of G1 and M phase durations is described in Materials and Methods. L.I. values (in C) and mitotic L.I. values (in D) are the mean ± S.E.M. (n ≥ 4). The P value indicated in C was obtained using the Linear Regression Model of the Prism software as indicated in Materials and Methods. Bars = 20 μm.

The results presented so far suggested that DYRK1A-dependent phosphorylation of T286 in Cyclin D1 might provide a means of regulating Cyclin D1-Cdk activity in RG progenitors. Indeed, in fibroblast cells the phosphorylation of Cyclin D1 by DYRK1A induces a dose-dependent increase in the duration of the G1 phase (Chen et al., 2013). Given the importance of Cyclin D-Cdk activity in cell cycle progression, we wanted to know whether the deficit of Cyclin D1 in Tg*Dyrk1a* RG progenitors was affecting G1 phase duration. To test this possibility, we estimated the duration of the total cell cycle and the length of the different cell cycle phases in telencephalic RG progenitors of E11.5 wild-type and Tg*Dyrk1a* embryos by assessing the accumulation of 5-ethynyl-2′deoxyuridine (EdU) *in vivo*, as described previously (Takahashi et al., 1993, Arai et al., 2011) (see schedule in Fig. 2A). The growth fraction (GF) of RG progenitors (nuclei in the VZ that do not express Tbr2 or Tuj1; Fig. 2B) reached the maximum labelling index value in both genotypes (Fig. 2C), indicating that all progenitors were cycling. However, total cell cycle duration (Tc) and S phase duration (Ts) calculated from the best fitted slope defined by the increasing labelling index values (Fig. 2C) were increased by 4.8 h and by 2.8 h, respectively, in the transgenic progenitors overexpressing DYRK1A (Fig. 2E). Duration of the G2 and M phases, measured by combining EdU labelling with staining for phospho-Histone 3 (pH3) to label cells in mitosis (Fig. 2D), was similar in both genotypes (Fig. 2E). In contrast, the duration of the G1 phase, derived by subtracting the duration of the S/G2/M phases from the Tc value, was 1.9 h longer in Tg*Dyrk1a* progenitors than in the wild-type. Thus, cell cycle lengthening in RG progenitors overexpressing DYRK1A results from an increased duration of the G1 and S phases. S phase durations in E11.5 wild-type and Tg*Dyrk1a* RG progenitors assessed by the IdU, BrdU double-labelling method described in Martynoga et al. (2005) (see Supplementary Fig. 4A–C) also showed that transgenic progenitors overexpressing DYRK1A have longer S phases than wild-type progenitors (Supplementary Fig. 4D). The increase in S phase duration calculated in this experiment (28% increase with respect to the wild-types) was similar to the increase calculated by assessing the accumulation of EdU (35%; Fig. 2E). However, we did find significant differences between the Ts values calculated in the two experiments (Fig. 2E and Supplementary Fig. 4D). This discrepancy should be taken into consideration when comparing cell cycle parameters assessed using different methodologies.

Together, these results indicate that a 1.5-fold increase in DYRK1A protein is sufficient to lower Cyclin D1 levels and alter cell cycle parameters of RG progenitors, lengthening G1 phase, consistent with the ability of DYRK1A to promote phosphorylation of T286 in Cyclin D1.

### 3.3 DYRK1A Modifies the Proportion of RG-derived Neurons and IPs in a Dosage-dependent Manner

**Fig. 3:**
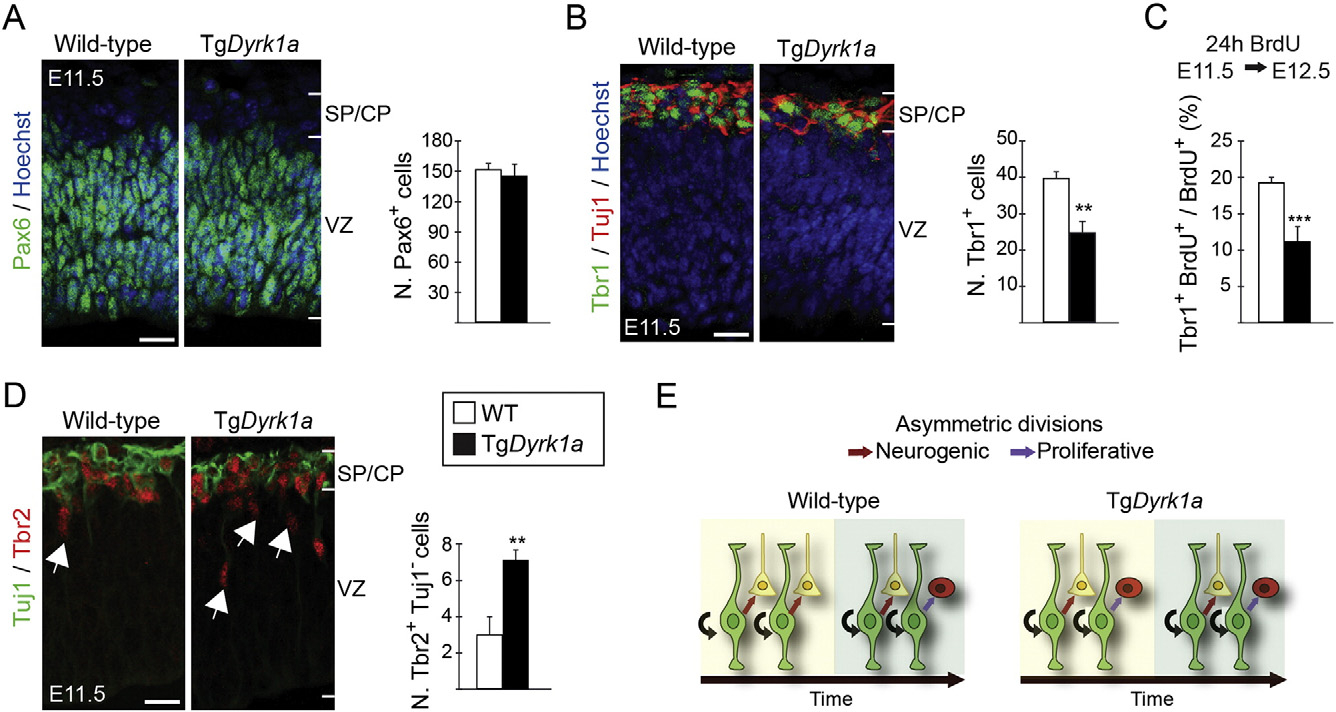
Impaired early neurogenesis and increased production of IPs in Tg*Dyrk1a* embryos. (A, B, D) Representative confocal images from coronal brain sections of E11.5 wild-type (WT) and Tg*Dyrk1a* embryos showing Pax6 expression (green in A), Tbr1 and Tuj1 expression (green and red in B, respectively) and Tuj1 and Tbr2 (green and red in C, respectively). Histograms show the numbers of Pax6^+^ radial glial progenitors (A), Tbr1^+^ neurons (B) and Tbr2^+^ intermediate progenitors; Tbr2^+^,Tuj1^−^ cells (arrows; D) in a 100 μm-wide column of the cortical wall. (C) Histogram showing neuronal production in the dorsal telencephalon of WT and Tg*Dyrk1a* embryos obtained from BrdU-injected females at E11.5 and harvested 24 h later. Values are the percentage of cells containing BrdU that express the neuronal marker Tbr1 and were obtained by counting BrdU^+^, Tbr1^+^ double immunofluorescent cells in a 100 μm-wide column of the cortical wall. Histogram values are the mean ± S.E.M. **P < 0.01, ***P < 0.001 (n = 3 in A, B, D; n = 4 in C). Bars = 20 μm. CP, cortical plate; SP, subplate; VZ, ventricular zone. (E) Scheme summarizing the cell count results in A to D. Radial glial progenitors (Pax6^+^ cells in green) in the dorsal VZ of both WT and Tg*Dyrk1a* embryos divide asymmetrically producing another Pax6 progenitor (black arrows) and a neuron (yellow cell) or an intermediate progenitor (red cell). In Tg*Dyrk1a* embryos the number of divisions producing intermediate progenitors increases at the expenses of the ones producing neurons.

Given the established link between G1 phase duration and the fate of the progenitor daughters (Calegari et al., 2005, Salomoni and Calegari, 2010), we then asked whether a moderate increase in G1 phase duration in RG progenitors is sufficient to affect their neurogenic potential. To this end, we counted the number of cells expressing neural markers in brain sections of E11.5 wild-type and Tg*Dyrk1a* embryos. The thickness of the dorsal telencephalic VZ at this stage and the number of RG progenitors (Pax6^+^ cells) were very similar in both genotypes (wild-type, 110.7 ± 3.19 μm; Tg*Dyrk1a*, 111.8 ± 3.72 μm and Fig. 3A). In contrast, Tg*Dyrk1a* embryos have fewer cells expressing the neuronal marker Tbr1 than wild-type embryos (Fig. 3B). This reduction was not due to increased cell death, since the number of apoptotic cells immunolabelled for active caspase3 was similar in both genotypes (cells in a 400 μm-wide column were 1.95 ± 0.16 and 1.96 ± 0.31 in wild-type and in Tg*Dyrk1a* embryos, respectively; see also Supplementary Fig. 5A). Thus, the deficit of differentiating neurons in the transgenic embryos likely results from impaired neuron production. Indeed, experiments in which the S phase marker BrdU was injected into pregnant females at E11.5 and embryos analysed 24 h later showed a 40% reduction in RG-derived neurons (proportion of BrdU^+^ cells expressing Tbr1) in Tg*Dyrk1a* embryos with respect to the wild-types (Fig. 3C). This reduction cannot be explained only by the moderate lengthening of the cell cycle observed (28% respect to the wild-types; Fig. 2E), suggesting a possible bias in the division mode of RG progenitors in the transgenic embryos.

During the neurogenic phase of neocortical development, RG self-renewing divisions produce either one neuron or one IP (Noctor et al., 2004, Haubensak et al., 2004, Miyata et al., 2004). The transition of a RG progenitor to an IP is associated with the downregulation of Pax6 and the upregulation of Tbr2 (Englund et al., 2005), which is a transcription factor required for IP specification and marks this progenitor type (Sessa et al., 2008). The Pax6–Tbr2 switch also takes place in RG-derived neurons, but the expression of Tbr2 in these cells is shut down as they move from the VZ to the cortical plate and start to express neuronal markers. However, at the onset of IP production, by E11.5, some neurons still have detectable levels of Tbr2 (Englund et al., 2005). Therefore, to estimate RG-derived IP production at this developmental stage, we counted the Tbr2^+^ cells that did not express the neuronal marker Tuj1 (arrows in Fig. 3D). The cell counts showed that Tg*Dyrk1a* embryos had more IPs than the wild-types (Fig. 3D). Together, these observations suggest that the deficit of early-born cortical neurons in the transgenic condition may result from an increased proportion of RG proliferative divisions at the expense of the neurogenic divisions (Fig. 3E). As progenitors undergoing proliferative divisions have longer S phases than the ones undergoing neurogenic divisions (Arai et al., 2011), the bias observed in the division mode of Tg*Dyrk1a* RG progenitors was consistent with the increased duration of the S phase in these progenitors (Fig. 2E and Supplementary Fig. 4).

**Fig. 4:**
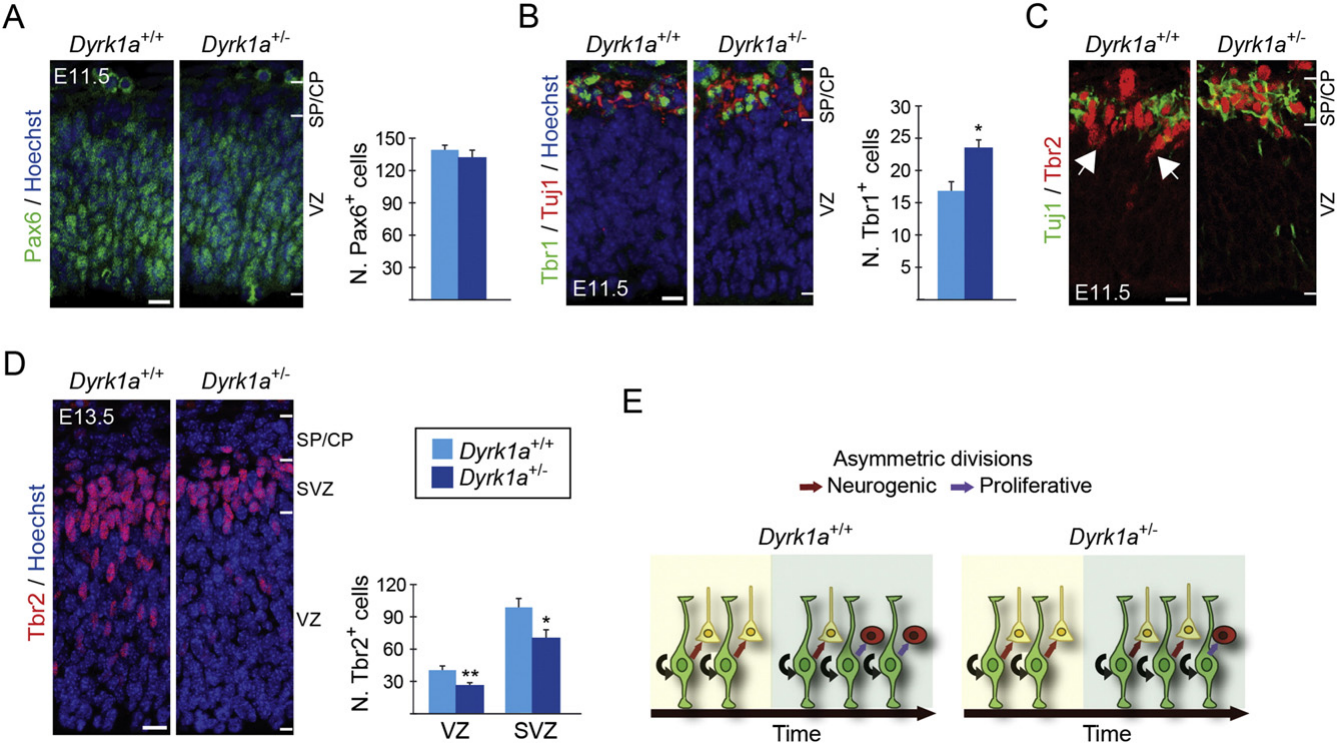
Increased early neurogenesis and decreased production of IPs in *Dyrk1a*^+/−^ embryos. (A to D) Representative confocal images from coronal brain sections of E11.5 (A to C) and E13.5 (D) *Dyrk1a*^+/+^ and *Dyrk1a*^+/−^ embryos showing Pax6 expression (A), Tbr1 and Tuj1 expression (B), Tbr2 and Tuj1 expression (C) and Tbr2 expression (D). Histograms show the numbers of Pax6^+^ radial glial progenitors (A), Tbr1^+^ neurons (B) and Tbr2^+^ intermediate progenitors (D) in a 100 μm-wide column of the cortical wall. Histogram values are the mean ± S.E.M. *P < 0.05, **P < 0.01 (n ≥ 3 in A to C; n = 4 in D). Bars = 20 μm. CP, cortical plate; SP, subplate; VZ, ventricular zone; SVZ, subventricular zone. (E) Scheme summarizing cell count results in A to D. Radial glial progenitors (Pax6^+^ cells in green) in the VZ of both *Dyrk1a*^+/+^ and *Dyrk1a*^+/−^ embryos divide asymmetrically producing another Pax6 progenitor (black arrows) and a neuron (yellow cell) or an intermediate progenitor (red cell). In *Dyrk1a*^+/−^ mutants the number of divisions producing neurons increases at the expenses of the ones producing progenitors.

If DYRK1A-induced degradation of Cyclin D1 is the mechanisms by which this kinase regulates G1 phase duration in RG progenitors and hence the fate of their daughter cells, lowering DYRK1A protein levels in these progenitors should also modify the proportion of neurons and IPs they produce. To test this prediction we did the same quantifications in embryos heterozygous for a *Dyrk1a* null mutation (*Dyrk1a*^+/−^ embryos) (Fotaki et al., 2002). The levels of DYRK1A protein in the telencephalon of E11.5 *Dyrk1a*^+/−^ embryos were reduced (around 50%) with respect to the levels in the *Dyrk1a*^+/+^ control littermates. Importantly, the levels of nuclear Cyclin D1 in *Dyrk1a*^+/−^ dorsal RG progenitors were significantly increased (Supplementary Fig. 5B and C). As in the Tg*Dyrk1a* gain-of-function model, there were no differences between genotypes in the number of RG progenitors (Pax6^+^ cells in the VZ; Fig. 4A). However, *Dyrk1a*^+/−^ embryos had more Tbr1-expressing neurons than *Dyrk1a*^+/+^ embryos (Fig. 4B). We could not detect any IP cells (Tbr2^+^, Tuj1^−^ cells) in E11.5 *Dyrk1a*^+/−^ embryos (Fig. 4C), indicating that IP production is impaired in this *Dyrk1a* mutant. To confirm this, we counted the number of Tbr2^+^ cells in *Dyrk1a*^+/+^ and *Dyrk1a*^+/−^ embryos two days later (at E13.5), an age where the germinal SVZ is well formed. The number of these cells was reduced in the VZ of *Dyrk1a*^+/−^ embryos (Fig. 4D), showing that indeed mutant RG progenitors produce fewer IPs than the controls. Accordingly, Dyrk1a+/- mutants had less IPs in the SVZ (Fig. 4D).

The opposing phenotypes of *Dyrk1a* gain- and loss-of-function mutant embryos strongly suggest that moderate variations in the levels of nuclear Cyclin D1 in RG progenitors bias their division mode, favouring asymmetric proliferative divisions when Cyclin D1 levels decrease and asymmetric neurogenic divisions when they increase (see schemes in Figs. 3E and 4E).

### 3.4 Triplication of *Dyrk1a* Alters Neuron Production and the Onset of Gliogenesis in the Dorsal Telencephalon

**Fig. 5:**
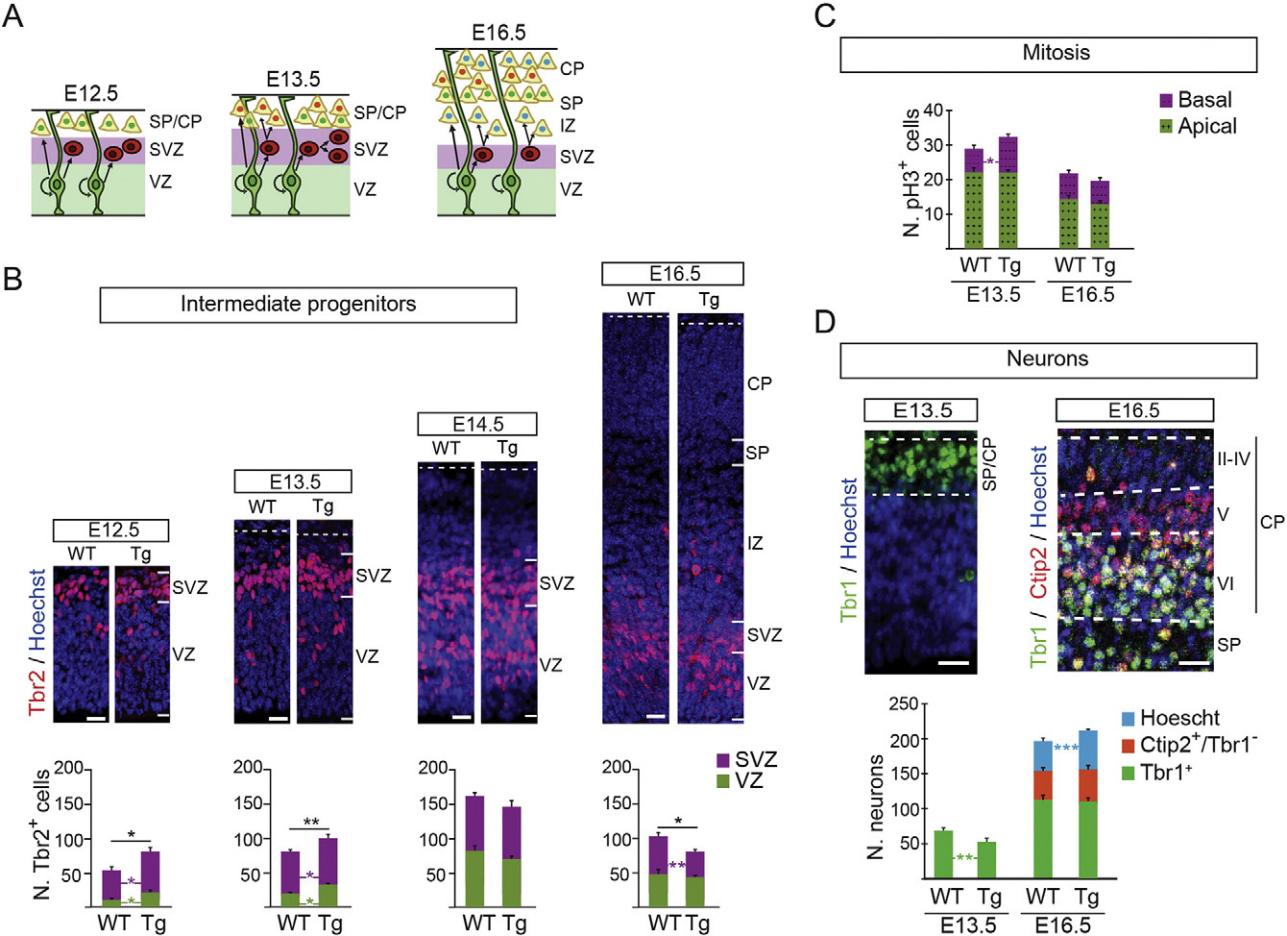
Tg*Dyrk1a* embryos show altered numbers of neurons and IPs along corticogenesis. (A) Scheme showing the cellularity of the ventricular (VZ) and subventricular (SVZ) germinal layers along the neurogenic phase of neocortical development and the division mode of the two main progenitor types in these layers; radial glial progenitors (in green) and intermediate progenitors (in red). Note that cortical neurons are generated following an inside–outside pattern and that most of the upper layer neurons (yellow neurons with blue nuclei) are produced around E16.5 by terminal divisions of intermediate progenitors. (B) Representative images of wild-type (WT) and Tg*Dyrk1a* (Tg) embryonic brain sections from different developmental stages immunostained for Tbr2 and nuclei labelled with Hoechst, and histograms showing the numbers of Tbr2^+^ progenitors (intermediate progenitors) in the VZ (green bars) and in the SVZ (purple bars) in a 100 μm-wide column of the cortical wall. (C) Histogram showing the number of pH3^+^ cells in the VZ (green bar; apical mitosis) and in the SVZ (purple bar; basal mitosis) in a 600 μm-wide column of the cortical wall. (D) Representative images of WT brain embryo sections of the indicated developmental stages immunostained for layer-specific markers, and histogram showing the numbers of total neurons (Tbr1^+^ cells) at E13.5, and the numbers of layer VI neurons (Tbr1^+^ cells), layer V neurons (Ctip2^+^, Tbr1^−^ cells) and layers II–IV neurons (Ctip2^−^, Tbr1^−^ Hoechst labelled cells) at E16.5 in a 100 μm-wide column of the cortical wall. Histogram values are the mean ± S.E.M. *P < 0.05, **P < 0.01, ***P < 0.001 (n ≥ 3). Bars = 20 μm. CP, cortical plate; IZ, intermediate zone; SP, Subplate.

The majority of neurons in the neocortex are generated in the SVZ from IPs (indirect neurogenesis) (Kowalczyk et al., 2009, Noctor et al., 2007; and scheme in Fig. 5A) and, therefore, a small variation in the number of these progenitors is expected to have a significant impact on neuron cellularity. Since the production of IPs is augmented in E11.5 Tg*Dyrk1a* embryos (Fig. 3D), and DYRK1A is expressed in the VZ and SVZ during all the neurogenic phase of cortical development (Supplementary Fig. 6), we counted the IPs generated from RG progenitors (Tbr2^+^ cells in the VZ; Fig. 5B) and the ones in the SVZ (Tbr2^+^ cells in the SVZ; Fig. 5B) in wild-type and Tg*Dyrk1a* embryos at different developmental stages. During early neurogenesis, until E14.5, the number of Tbr2 progenitors in the VZ and SVZ increased in both wild-type and transgenic embryos (Fig. 5A and B). However, Tg*Dyrk1a* embryos had more Tbr2 progenitors in both germinal regions at E12.5 and E13.5 (Fig. 5B).

Contrary to RG progenitors that divide in the VZ, IPs divide in the SVZ (Noctor et al., 2004, Haubensak et al., 2004, Miyata et al., 2004; and scheme in Fig. 5A). The number of pH3^+^ cells in the telencephalic ventricular surface (apical mitosis) was similar in wild-type and Tg*Dyrk1a* embryos at E13.5 and E16.5 (Fig. 5C), indicating that during neurogenesis the number of RG divisions was not altered in *Dyrk1a* transgenic embryos. Accordingly, there were no differences between genotypes in the numbers of RG progenitors during neurogenesis (Pax6^+^ cells in a 100 μm-wide column were: wild-type, 127.11 ± 7.56; Tg*Dyrk1a*, 133.83 ± 6.23 at E13.5 and wild-type, 72.61 ± 3.98; Tg*Dyrk1a*, 80.53 ± 5.91 at E16.5). However, the number of pH3^+^ cells in the SVZ (basal mitosis) was significantly increased in E13.5 Tg*Dyrk1a* embryos, which correlated with the increased number of IPs in the SVZ observed at this stage (Fig. 5C).

As neurogenesis proceeds, IPs become the main source of neocortical neurons (Kowalczyk et al., 2009; and Fig. 5A). To evaluate how the increased number of IPs affects neuron production in the transgenic condition, we counted all differentiating neurons in the dorsal telencephalon of wild-type and Tg*Dyrk1a* embryos at E13.5 using the neuronal marker Tbr1. The number of Tbr1^+^ cells in the transgenic embryos was still lower at this developmental stage (Fig. 5D), but the reduction (15%), was less severe than in E11.5 (40%). This suggests that indirect neurogenesis is higher in Tg*Dyrk1a* embryos than in the wild-types, partially compensating for the deficit of RG-derived neurons (Fig. 3B and C). To provide evidence for this, we labelled cells in S-phase with BrdU at E13.5 and estimated the proportion of these cells that exited the cell cycle 24 h later by doing double immunostaining with BrdU and Ki67 antibodies. As shown in Supplementary Fig. 7A, cell cycle exit rates were similar in the telencephalon of wild-type and Tg*Dyrk1a* embryos, indicating that indeed indirect neurogenesis at this stage is reducing the deficit of early-born RG-derived neurons in embryos with 3 copies of *Dyrk1a*. Then we asked whether the advanced production of IPs in these embryos (Figs. 3D and 5B) alters the fate of the neurons they produce. The results of a birthdate experiment performed by injecting BrdU into pregnant females at E13.5, when the production of layer V callosal projection neuron peaks (Molyneaux et al., 2007), indicated that the differentiating program in the dorsal telencephalon of Tg*Dyrk1a* embryos is slightly advanced (Supplementary Fig. 7B and C), paralleling the advanced production of IPs.

In wild-type embryos, during the late phase of cortical neurogenesis, by E16.5, the production of IPs (Tbr2^+^ cells in the VZ; Fig. 5B) decreases and the number of IP terminal divisions in the SVZ increases (Kriegstein and Alvarez-Buylla, 2009; and scheme in Fig. 5A). IP production in the VZ of Tg*Dyrk1a* embryos was normal at E14.5 and E16.5 (green rectangles in Fig. 5B). The number of IPs in the SVZ of these embryos was also normal at E14.5 but decreased at E16.5 (purple rectangles in Fig. 5B). This decrease did not result from increased apoptosis, since the number of cells in E14.5 embryos that expressed the active form of caspase3 was similar in both genotypes (cells in a 1400 μm-wide column were 7.9 ± 1.26 and 9.5 ± 1.50 in wild-type and Tg*Dyrk1a* embryos, respectively). Since the number of mitoses in the SVZ of E16.5 Tg*Dyrk1a* and wild-type embryos was similar (Fig. 5C), the decreased number of SVZ IPs observed in Tg*Dyrk1a* embryos at this stage (Fig. 5B) could result from increased terminal divisions leading to an advanced exhaustion of the IP pool. If this were the case, the production of neurons in Tg*Dyrk1a* embryos should increase between E14.5 and E16.5. To asses this, we counted cells in the cortical layers defined by the layer-specific markers Tbr1 and Ctip2 at E16.5 (Molyneaux et al., 2007). As predicted, the number of neurons in the external layers (defined by the lack of Tbr1 and Ctip2 expression) was augmented in Tg*Dyrk1a* embryos (Fig. 5D). At this stage, Tbr1 is expressed in layer VI early-born neurons (Bulfone et al., 1995) and in newborn upper layer neurons that are migrating to their final position and still expressed this transcription factor. The deficit of Tbr1^+^ neurons observed in E13.5 Tg*Dyrk1a* embryos was no longer evident at E16.5 (Fig. 5D), which is in accordance with more neurons being produced in the transgenic embryos at this stage. The number of layer V neurons (cells expressing Ctip2) was not affected by the overexpression of DYRK1A (Fig. 5D). This, together with the birthdate data shown in Supplementary Fig. 7C, suggests that Ctip2 neurons in the *Dyrk1a* embryos are produced earlier in development but at normal rates. In summary, our results show that a 1.5-fold increase in DYRK1A protein levels disturbs the number of neocortical neurons that are generated through development by direct and indirect neurogenesis.

As neurogenesis progresses and the length of the G1 phase increases, the levels of Cyclin D1 in dorsal VZ cells progressively decrease, and by the end of the neurogenic phase, by E18.5, Cyclin D1 immunolabelling in the ventricular proliferative region was very faint (Supplementary Fig. 8A). Similar to the situation at the onset of neurogenesis (Fig. 1B and C), the levels of nuclear Cyclin D1 inversely correlated to the levels of DYRK1A in E13.5 embryos (Supplementary Figs. 6 and 8B). In contrast and despite DYRK1A being still present in the VZ of E16.5 embryos (Supplementary Fig. 6), the levels of nuclear Cyclin D1 in Tg*Dyrk1a* VZ progenitors were normal (Supplementary Figs. 6A and 8C). Consistent with published data (Glickstein et al., 2009), we only detected a few cells expressing high Cyclin D1 levels in the SVZ. Therefore, our expression data and the phenotype of Tg*Dyrk1a* transgenic embryos indicate that the altered production of upper layer neurons observed in these embryos does not result from the regulatory action of DYRK1A on Cyclin D1 degradation.

**Fig. 6:**
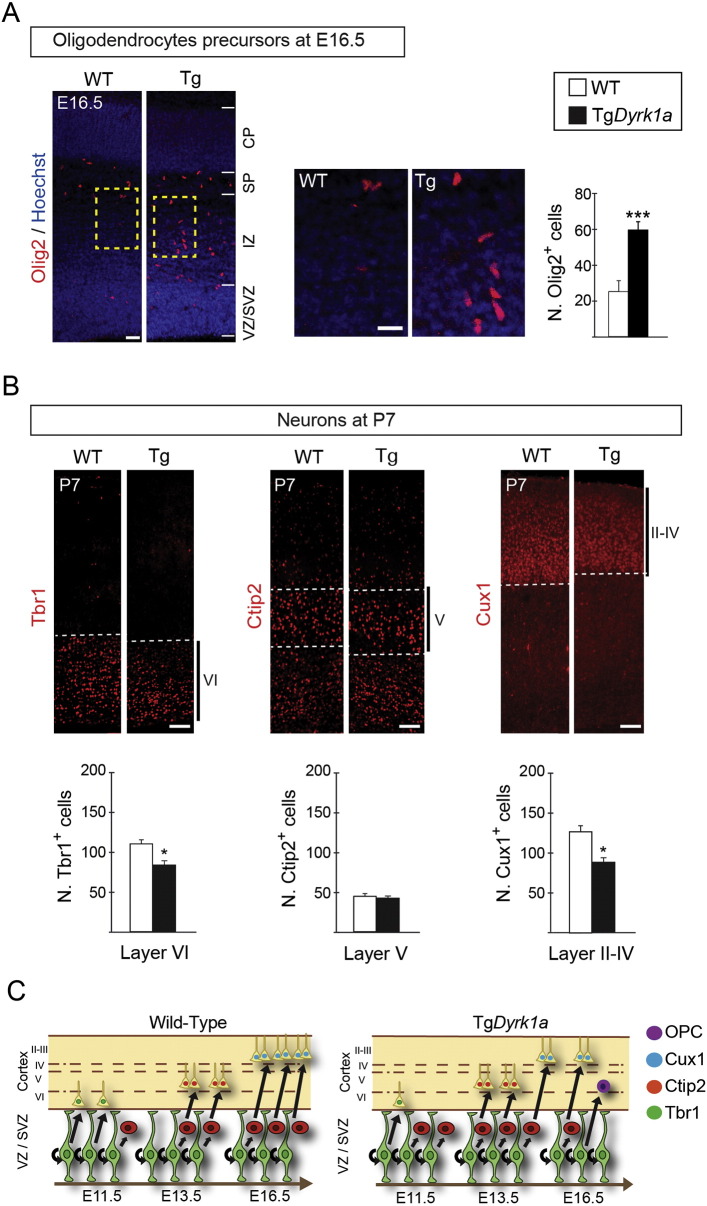
The Tg*Dyrk1a* model shows an advanced production of oligodendrocyte progenitors and decreased neuronal cellularity in specific cortical layers. (A) Representative coronal sections from E16.5 wild-type (WT) and Tg*Dyrk1a* (Tg) brains immunostained for Olig2 and nuclei labelled with Hoescht. Yellow dashed rectangles indicate the region magnified in the images on the right. Histogram shows the number of Olig2^+^ cells in a 300 μm-wide column of the cortical wall. (B) Representative coronal sections from P7 WT and Tg brains immunostained for Tbr1 (left), Ctip2 (middle) or Cux1 (right), and histograms showing the number of layer VI Tbr1^+^ neurons, layer V Ctip2^+^ neurons and layers II–IV Cux1^+^ neurons in a 100 μm-wide column of the cortical wall. Values are the mean ± S.E.M. *P < 0.05, ***P < 0.001 (n ≥ 3). Bars = 50 μm. CP, cortical plate; SP, subplate; VZ, ventricular zone; SVZ, Subventricular zone. (C) Schemes showing the birth time of Tbr1 neurons, Ctip2 neurons, Cux1 neurons and oligodendrocyte precursor cells (OPCc) in the telencephalon of a WT and a Tg embryo and the progenitor type (radial glial (green) or intermediate (red) progenitor) that produces these cells. The deficits of early-born (Tbr1^+^) neurons and late born (Cux1^+^) neurons in transgenic animals result, respectively, from the decreased neuronal production during early neurogenesis and from the premature exhaustion of the intermediate progenitor pool during late neurogenesis. Note that the generation of OPCs is advanced in Tg embryos.

The generation of macroglial cells in the mouse dorsal telencephalon begins around birth when VZ progenitors lose their capacity to generate neurons and become gliogenic (Kriegstein and Alvarez-Buylla, 2009). Immunolabelling for Cyclin D1 revealed the presence of a population of cells expressing high levels of the protein in the region above the SVZ, the intermediate zone, of E16.5 Tg*Dyrk1a* embryos that was almost absent in the wild-types (Supplementary Fig. 8C). Most of these cells also expressed Platelet-derived growth factor receptor-alpha (PDGFRα) (Supplementary Fig. 8D), which is a marker of oligodendrocyte precursors (Rowitch, 2004). In accordance, Tg*Dyrk1a* embryos at this stage had an increased number of precursors expressing the oligodendrocytic marker Olig2 (Rowitch, 2004) (Fig. 6A). These results indicate that Tg*Dyrk1a* RG progenitors acquire their capacity of producing glial cells before the wild-types (Fig. 6C).

### 3.5 Postnatal Tg*Dyrk1a* Mice Exhibit an Altered Proportion of Neocortical Projection Neurons

To estimate the impact of the neurogenic defects observed in Tg*Dyrk1a* embryos, we counted the number of neurons expressing layer-specific markers in wild-type and Tg*Dyrk1a* mice at P0, just after the end of neurogenesis, and at P7, when radial migration has ended and projection neurons are in their final layer position (Miller, 1988). At these developmental stages, Tbr1 expression in the neocortex is almost restricted to layer VI neurons (Bulfone et al., 1995), which are mainly generated between E11.5 and E13.5 (Molyneaux et al., 2007). According to the neuron deficit observed in Tg*Dyrk1a* embryos during this period (Figs. 3B and 5D), the number of layer VI Tbr1^+^ neurons in transgenic animals was lower than in the wild-types at both P0 and P7 (Fig. 6B and Supplementary Fig. 9A). In contrast, the number of layer V subcerebral neurons that express high levels of Ctip2 (Arlotta et al., 2005) was normal in Tg*Dyrk1a* postnatal animals (Fig. 6B and Supplementary Fig. 9B), which is in accordance with the normal numbers of layer V Ctip2 neurons observed in E16.5 *Dyrk1a* transgenic embryos (Fig. 5D). Superficial cortical projection neurons are mostly produced by SVZ IPs and express the transcription factors Mef2c (Leifer et al., 1993) and Cux1 (Nieto et al., 2004). The number of layers II–IV Mef2c^+^ neurons at P0 (Supplementary Fig. 9B) and of Cux1^+^ neurons at P7 (Fig. 6B) was significantly lower in Tg*Dyrk1a* animals than in the wild-types. This correlated with the decrease in IP cell numbers displayed by transgenic embryos from E14.5 to E16.5 (Fig. 5B). Moreover, Tbr1^+^, Ctip2^+^ and Cux1^+^ neurons in P7 Tg*Dyrk1a* animals had a normal layer distribution (Fig. 6B), indicating that the overexpression of DYRK1A did not affect radial migration of cortical differentiating neurons.

In summary, the results presented so far show that a 1.5-fold increase in DYRK1A protein levels diminishes neuron production rates at the beginning (by E11.5) and the end (by E16.5) of dorsal cortical neurogenesis. The deficits in early-born neurons and late-born neurons in the DYRK1A overexpressing model can be explained, respectively, by the impaired production of RG-derived neurons and the early exhaustion of the IP pool (see scheme in Fig. 6C).

### 3.6 Normalization of *Dyrk1a* Gene-dosage Restores Cyclin D1 Protein Levels and the Neurogenic Potential of RG Progenitors in Ts65Dn Embryos

**Fig. 7:**
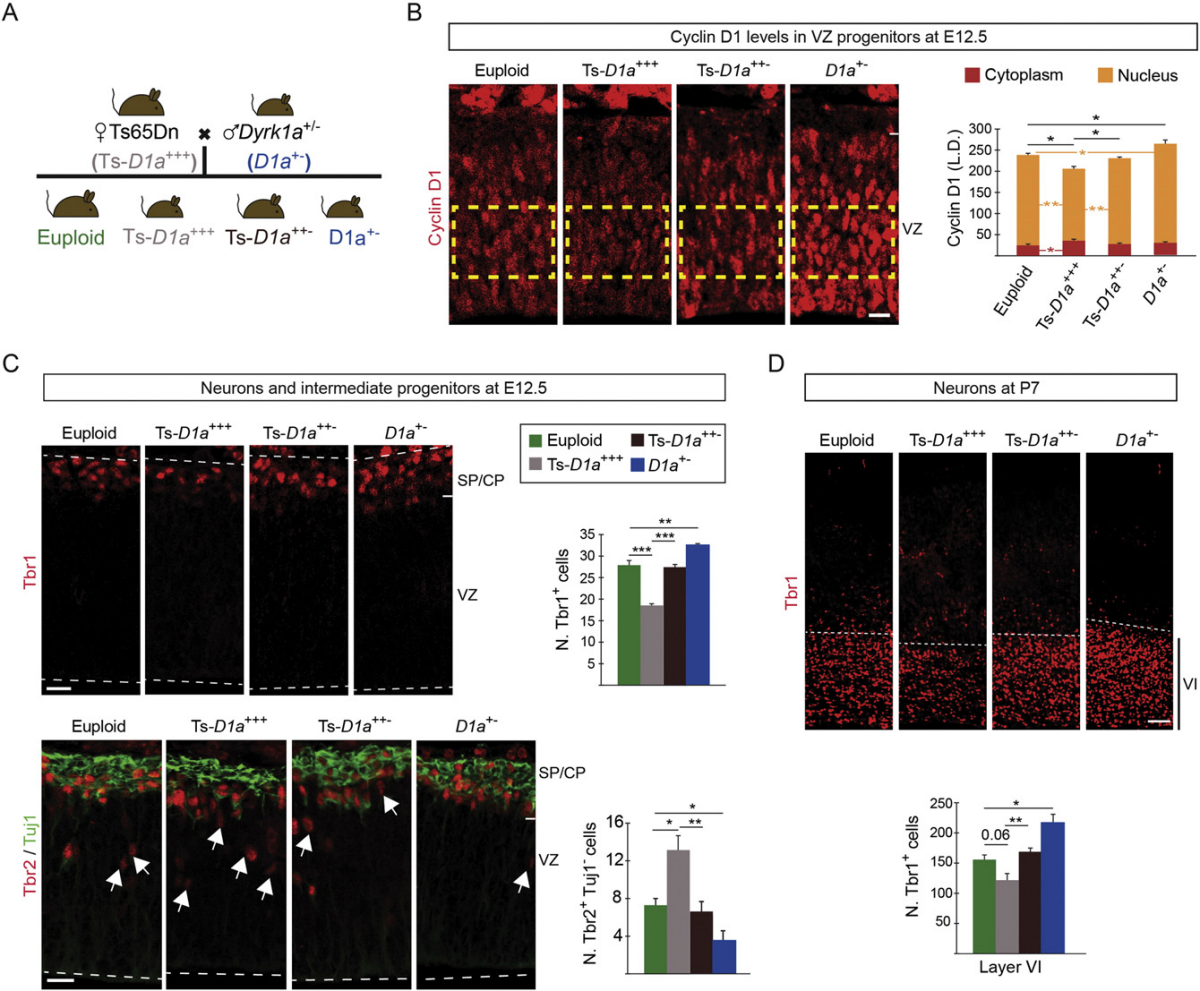
Trisomy of *Dyrk1a* alters early cortical neurogenesis in the Ts65Dn Down syndrome model. (A) Scheme of the crosses to generate Ts65Dn mice (Ts-*D1a*^+++^), Ts65Dn mice with 2 functional copies of *Dyrk1a* (Ts-*D1a*^++−^), euploid mice monosomic for *Dyrk1a* (*D1a*^+/−^) and control (euploid) mice. (B–D) Representative brain coronal sections obtained from E12.5 embryos of the indicated genotypes and immunolabelled for Cyclin D1 (B), Tbr1 (C), or Tbr2 and Tuj1 (C), or from P7 postnatal mice of the indicated genotypes immunolabelled for Tbr1 (D). Histogram in B shows the labelling density (L.D.) of Cyclin D1 fluorescence signal in the nucleus and the cytoplasm of radial glial progenitors of the ventricular zone (VZ) region indicated by the yellow rectangle and calculated as shown in Supplementary Fig. 2C and D. Histograms in C and D show the numbers of Tbr1^+^ neurons (C and D) and Tbr2^+^ intermediate progenitors; Tbr2^+^,Tuj1^−^ cells (C) in a 100 μm-wide column of the cortical wall. Histogram values are the mean ± S.E.M. *P < 0.05, **P < 0.01, ***P < 0.001 (n ≥ 3). Bars = 20 μm in B and C, and 50 μm in D. CP, cortical plate; SP, subplate.

Similar to the Tg*Dyrk1a* model, VZ progenitors of the dorsal telencephalon in trisomic Ts65Dn embryos have longer cell cycle duration than euploid progenitors. Importantly, these embryos show reduced neuron production during the early phase of corticogenesis and a transient increase in the numbers of Tbr2^+^ IPs and SVZ dividing progenitors (cells in mitosis) at later stages. As a consequence, the growth of the cortical wall is delayed in trisomic Ts65Dn embryos (Chakrabarti et al., 2007). The similarities between the Ts65Dn model and the Tg*Dyrk1a* model reported here, suggest that the triplication of *Dyrk1a* contributes to the early neurogenic cortical defects occurring prenatally in the Ts65Dn model. To investigate this, we first checked Cyclin D1 protein levels in total telencephalic extracts of E11.5 Ts65Dn embryos, which contain 1.5-fold more DYRK1A protein than the extracts from euploid embryos. Importantly, Cyclin D1 levels were decreased in Ts65Dn embryos (Supplementary Fig. 10). Next, we checked whether normalization of *Dyrk1a* gene-dosage in the trisomic embryos could normalize Cyclin D1 levels in dorsal RG progenitors. For this, we estimated the levels of Cyclin D1 in the nucleus and cytosol of RG progenitors in embryo brain sections obtained from crosses between Ts65Dn females and *Dyrk1a*^+/−^ males (Fig. 7A) by using the same analysis we did before in Tg*Dyrk1a* embryos (Supplementary Fig. 2C and D). By E12.5, one day after the onset of neurogenesis in the dorsal telencephalon, Cyclin D1 protein levels in Ts65Dn RG progenitors were lower than in euploid progenitors (Fig. 7B). Moreover, Cyclin D1 content in the nuclei of Ts65Dn progenitors diminished to similar extents as in Tg*Dyrk1a* progenitors (Figs. 1C and 7B). These data indicate that nuclear levels of Cyclin D1 in Ts65Dn apical progenitors are controlled by DYRK1A. Accordingly, the genetic normalization of *Dyrk1a* dosage in Ts65Dn progenitors increased the amount of total and nuclear Cyclin D1 to euploid levels (Fig. 7B). As expected by the result presented in Supplementary Fig. 5B, RG progenitors in *Dyrk1a*^+/−^ littermate embryos had more Cyclin D1 in their nuclei than in euploid embryos (Fig. 7B).

In Ts65Dn embryos, progenitors of the telencephalic VZ have longer cell cycle duration due to a lengthening of the G1 and S phases (Chakrabarti et al., 2007). If G1 phase lengthening biases the type of RG self-renewing divisions, as we propose for Tg*Dyrk1a* progenitors (Fig. 3E), Ts65Dn RG progenitors should produce fewer neurons and more IPs. To investigate this possibility we counted apical progenitors (Pax6^+^ cells), neurons (Tbr1^+^ cells) and IPs (Tbr2^+^ cells that do not express the neuronal marker Tuj1) in E12.5 brain sections of the four genotypes resulting from the crosses depicted in Fig. 7A. As in the Tg*Dyrk1a* model, the thickness of the dorsal VZ and the numbers of Pax6^+^ progenitors in radial columns were similar in euploid and Ts65Dn embryos (wild-type, 135.8 ± 3.06 μm; Ts65Dn, 131.3 ± 3.70 μm; Supplementary Fig. 11). Moreover, Ts65Dn progenitors produced fewer neurons (Supplementary Fig. 11A and Fig. 7C) and more IPs than euploid progenitors (Fig. 7C). The phenotype in Ts65Dn embryos reported here is consistent with the reduced production of early-born neurons and the increased number of Tbr2^+^ progenitors in the SVZ observed at later developmental stages in these embryos (Chakrabarti et al., 2007). Importantly, both neuron and IP numbers were normal in Ts65Dn embryos with normal *Dyrk1a* gene-dosage. In accordance with the data shown in Fig. 4B and D, *Dyrk1a*^+/−^ littermate embryos showed increased numbers of neurons and decreased number of Tbr2^+^ progenitors (Fig. 7C). This data indicates that at the onset of cortical neurogenesis triplication of *Dyrk1a* in Ts65Dn RG progenitors increases the production of IPs at the expense of neurons.

The decreased early neurogenesis in Ts65Dn embryos leads to a reduction in the number of layer VI Tbr1^+^ neurons in Ts65Dn postnatal animals (Chakrabarti et al., 2007, Chakrabarti et al., 2010). Ts65Dn animals resulting from crosses between trisomic mice and *Dyrk1a*^+/−^ mice (Fig. 7A) had fewer Tbr1 neurons than euploid animals (Fig. 7D). Importantly, the normalization of *Dyrk1a* gene-dosage in the trisomic embryos also restored the number of postnatal layer VI Tbr1 neurons (Fig. 7D). Similar to postnatal Tg*Dyrk1a* animals, Ts65Dn mice had normal numbers of layer V Ctip2^+^ neurons but decreased numbers of neurons in the external layers. This defect was reverted by decreasing *Dyrk1a* gene-dosage to normal levels (Supplementary Fig. 12), suggesting that the overexpression of DYRK1A also impacts late cortical neurogenesis in the trisomic Ts65Dn model.

In summary, these data show that increased *Dyrk1a* gene-dosage in RG Ts65Dn progenitors diminishes the production of early-born neurons in the developing neocortex and suggest that *Dyrk1a*-mediated degradation of Cyclin D1 is likely the basis of the altered cell cycle parameters observed previously in apical Ts65Dn progenitors.

## 4 Discussion

In the present study we show that a 1.5-fold increase in DYRK1A protein levels in NSCs of the mouse developing cerebral cortex caused by trisomy of the *Dyrk1a* gene lengthens the cell cycle and decreases the production of RG-derived neurons. Three key observations indicate that the overexpression of DYRK1A in RG progenitors favours RG asymmetric proliferative divisions at the expense of neurogenic divisions: (1) During the early phase of cortical neurogenesis, when the majority of neocortical neurons are generated in the VZ by RG-asymmetric neurogenic divisions (Kowalczyk et al., 2009), the number of IPs (Tbr2^+^ cells) that delaminate from the VZ surface and migrate to the basal surface increases in Tg*Dyrk1a* embryos whilst the number of neurons (Tbr1^+^ cells) decreases; (2) the number of RG progenitors (Pax6^+^ cells) and the number of mitoses in the apical surface of the ventricle in Tg*Dyrk1a* embryos are normal during corticogenesis and (3) the number of IPs in the SVZ of Tg*Dyrk1a* embryos increased during the growing phase of this germinal layer. Moreover, the fact that haploinsufficient *Dyrk1a*^+/−^ embryos produce fewer IPs and more neurons during early corticogenesis indicates that the effect of DYRK1A on the division mode of RG progenitors is dosage-dependent. In both Tg*Dyrk1a* and *Dyrk1a*^+/−^ mouse models, this defect leads to an altered number of projection neurons in the most internal layer of the neocortex.

The *in vivo* cell cycle parameters measured in RG progenitors showed that DYRK1A overexpression specifically affects the duration of the G1 and S cell cycle phases. Cyclin D1 has been shown to be rate limiting for the G1-to-S phase transition (Resnitzky et al., 1994). In the three mouse models used in this work with an imbalanced dosage of *Dyrk1a*, the levels of Cyclin D1 and DYRK1A proteins during the early phase of cortical neurogenesis were inversely correlated, indicating that DYRK1A might induce proteolytic degradation of Cyclin D1 in RG progenitors *in vivo*, as it does in some cultured mammalian cells (Chen et al., 2013, Yabut et al., 2010), which is consistent with the ability of DYRK1A to phosphorylate T286 in Cyclin D1. Whilst DYRK1A has been shown to increase both p21^CIP1^ (Park et al., 2010) and p27^KIP1^ levels (Soppa et al., 2014) in cell culture models we observed no change in the levels of these proteins in Tg*Dyrk1a* embryos. Thus changes in Cyclin D1, and not p21^CIP1^ or p27^KIP1^, are most likely to be mediating the effects of DYRK1A.

DYRK1B, the closest member to DYRK1A in the DYRK family of protein kinases (Aranda et al., 2011), also phosphorylates Cyclin D1 at T286 (Ashford et al., 2014) and its overexpression induces cell cycle arrest (Ashford et al., 2014). Data obtained in transcriptome analysis of different cell types of the mouse embryo by deep sequencing shows that the expression of *Dyrk1b* in dorsal telencephalic RG progenitors is very low compared to the expression of *Dyrk1a* in these cells (Aprea et al., 2013; GEO GSE51606), indicating that DYRK1A is likely to be the only DYRK kinase involved in the regulation of Cyclin D1 turnover in cortical RG progenitors.

During late corticogenesis, by E16.5, Cyclin D1 protein levels in Tg*Dyrk1a* RG progenitors were normal despite the fact that the levels of DYRK1A in the dorsal telencephalon were still augmented around 50%. Our Cyclin D1 expression data indicates that the levels of Cyclin D1 in wild-type cortical RG progenitors progressively decrease during neurogenesis, paralleling the increased duration of G1 phase (Takahashi et al., 1995). Thus, it is possible that nuclear Cyclin D1 during late neurogenesis is not regulating progression through G1 and, therefore, their levels are not subject to cell cycle regulation.

We show here that IPs express DYRK1A and that at late corticogenesis the pool size of this type of progenitor is significantly diminished in Tg*Dyrk1a* embryos. Consistent with our Cyclin D1 expression data, it has been shown that cortical IPs do not express Cyclin D1 but instead express Cyclin D2 (Glickstein et al., 2009). Thus, the early exhaustion of IPs in Tg*Dyrk1a* embryos and the consequent deficit of late-born neurons observed in this transgenic model at postnatal stages, could be caused by the dysregulation of a DYRK1A-mediated function other than Cyclin D1 degradation or could be secondary to the advanced production of these progenitors. Given that the neurogenic to gliogenic switch in Tg*Dyrk1a* RG progenitors is also advanced, the most plausible explanation for the late neurogenic defects observed in Tg*Dyrk1a* embryos is that all the neurogenic program in these embryos is slightly altered due to the lengthening of the G1 cell cycle phase that occurs at the beginning of the neurogenic phase of cortical development, which in the short-term leads to more IPs and less neurons.

Our results show a correlation between the output of RG asymmetric divisions and the duration of the G1 cell cycle phase in RG progenitors. This is in line with the accepted idea that the time that a RG progenitor spends in G1 determines the fate of their daughters (Dehay and Kennedy, 2007, Gotz and Huttner, 2005, Salomoni and Calegari, 2010); this is supported by experiments showing a significant effect on cortical neurogenesis by shortening the duration of the G1 phase *via* forced expression of Cyclin D1 or Cyclin D1/Cdk4 (Lange et al., 2009, Pilaz et al., 2009). The output of shortening G1 phase duration in the short-term (24 h–48 h) is a decrease in neuron production and a concomitant increase in IPs in the VZ and the SVZ, which is the same effect that we have observed at the beginning of neurogenesis in Tg*Dyrk1a* embryos. Manipulation of the G1 phase in the experiments reported in Lange et al. (2009) and Pilaz et al. (2009) took place during mid-corticogenesis (by E13–E14), which implies that the progenitors are intrinsically different and are exposed to different environmental cues than progenitors at earlier stages. This could explain why an increase in G1 phase duration at the beginning of neurogenesis and a decrease at later stages have similar outputs in the short-term.

In addition to a longer G1 phase, Tg*Dyrk1a* apical progenitors had a longer S phase. It has been reported that the length of the S phase in cortical progenitors is significantly shorter in progenitors committed to produce neurons than in those undergoing self-expanding divisions (Arai et al., 2011). Therefore, the lengthening of S phase in Tg*Dyrk1a* RG progenitors could be secondary to the increased number of RG asymmetric proliferative divisions producing IPs due to the action of DYRK1A on G1. The fact that expanding progenitors spend more time in S phase than those producing neurons has been interpreted as a need of these progenitors to guarantee high fidelity DNA replication and repair (Arai et al., 2011). In the early stages of neural system development, the repair of DNA double-strand breaks in proliferating cells occurs *via* homologous recombination (Orii et al., 2006). In addition to its Cdk-associated activity, nuclear Cyclin D1 promotes homologous recombination by recruiting RAD51 and BRCA2 to the DNA double-strand breaks (Jirawatnotai et al., 2011). Thus, it is possible that Tg*Dyrk1a* progenitors with limited amounts of Cyclin D1 spend more time in S phase to complete DNA replication.

DYRK1A regulates the activity of several signalling pathways (Aranda et al., 2011) including the calcium/calcineurin/NFATc pathway. In DS this pathway could be inhibited due to a functional interaction of *DYRK1A* with *RCAN1* (another chromosome 21 gene also known as *DSCR1*) on the activity of NFATc transcription factors (Arron et al., 2006). Recently, it has been reported that moderate overexpression of DYRK1A and RCAN1 in VZ cells achieved by *in utero* electroporation delays neurogenesis in the mouse dorsal telencephalon and that this effect requires the action of these two proteins on NFATc activity (Kurabayashi and Sanada, 2013). The phenotype observed here in Tg*Dyrk1a* embryos indicates that a 1.5-fold increase in DYRK1A protein levels in cortical progenitors reduces significantly the production of early-born neurons. We have measured the relative levels of *Rcan1* transcripts and protein isoforms, Rcan1-1 and Rcan1-4 (Davies et al., 2007), in the telencephalon of Tg*Dyrk1a* and wild-type littermate embryos at the onset of neurogenesis. In accordance with the *Rcan1* genetic complement in these embryos we did not find differences between genotypes (Supplementary Fig. 13). As the expression of the *Rcan1-4* transcript is induced by calcineurin-NFATc signalling (Yang et al., 2000), our Rcan1 expression data indicate that NFATc activity is not significantly perturbed in Tg*Dyrk1a* progenitors. *Rcan1* is in the chromosomal region that is triplicated in the Ts65Dn model (Haydar and Reeves, 2012). Thus, the fact that genetic normalization of *Dyrk1a* dosage in trisomic Ts65Dn embryos is sufficient to normalize the production of early-born cortical neurons in these embryos shows that *Dyrk1a* is the only gene in the triplicated region responsible for the phenotype.

Our results showing that nuclear levels of Cyclin D1 are reduced in the dorsal telencephalon of Ts65Dn embryos and that genetic normalization of *Dyrk1a* dosage restores Cyclin D1 to normal levels in the trisomic embryos suggests that the action of DYRK1A on Cyclin D1 turnover contributes to the altered cell cycle parameters observed previously in the VZ of these embryos (Chakrabarti et al., 2007). These results together with the decreased number of neurons and the concomitant increase in IPs observed here at the onset of cortical neurogenesis in Ts65Dn embryos indicate that DYRK1A-mediated dysregulation of Cyclin D1 levels in RG progenitors is the cause of the deficit of early-born cortical neurons and contributes to the expansion of the SVZ observed previously in the Ts65Dn model (Chakrabarti et al., 2007). Based on this, and the observation that postnatal Tg*Dyrk1a* and Ts65Dn mice have a similar deficit of neurons in their neocortices and that the deficit in both external and internal cortical neurons in the trisomic Ts65Dn mouse is restored by normalizing *Dyrk1a* gene-dosage, we hypothesise that DYRK1A is contributing to the delayed growth of the cortical wall previously observed in the Ts65Dn model (Chakrabarti et al., 2007). Future experiments should aim to confirm this hypothesis.

Cell cycle defects (Contestabile et al., 2007) and a deficit of neurons (Larsen et al., 2008, Schmidt-Sidor et al., 1990) have been observed in the cerebral cortex of DS foetuses. Given that fibroblast cells from DS individuals have less Cyclin D1 and enlarged G1 phases due to the overexpression of DYRK1A (Chen et al., 2013), and that the role of DYRK1A on brain development and growth is conserved across evolution (Tejedor and Hammerle, 2011), it is likely that the overexpression of DYRK1A in DS also increases G1 phase duration in RG progenitors of the developing neocortex thereby changing their neurogenic potential and final neuronal output.

DYRK1A has been proposed as a therapeutic target to ameliorate the cognitive deficit associated with DS (Becker and Sippl, 2011); indeed, treatment with epigallocatechin-gallate, which can inhibit DYRK1A, has been used successfully in DS mouse models and in a pilot study with young DS individuals to improve long-term memory (De la Torre et al., 2014). Our results show that the effect of DYRK1A on Cyclin D1 turnover in RG progenitors is dosage-dependent and that the deleterious effect of low levels of nuclear Cyclin D1 on DS cortical neurogenesis begins at early stages of prenatal development. These results should inform the design of prenatal therapeutic interventions aimed at counterbalancing the deleterious effect of *DYRK1A* triplication on cortical circuitry formation in DS. Finally, since mutations in the human *DYRK1A* gene in heterozygosity cause primary microcephaly (Courcet et al., 2012) and autism (O'Roak et al., 2012), the phenotype observed here in the *Dyrk1a*^+/−^ mouse model also provides insights into the aetiology of the neurological alterations associated with haploinsufficient mutations in the *DYRK1A* gene.

## Conflict of Interest

The authors declare no competing financial interests.

## Authors' Contributions

MLA conceived the project. MJB, SN, PAL and SJC designed the experiments and analysed the data. SN, JA, PAL, ALA, and DO performed the experiments. JMD generated the mBACtgDyrk1a mouse model. SN, MJB and SJC interpreted the data. SN prepared the figures and MLA wrote the manuscript.
