# Supplementary material for: DYRK1A-mediated Cyclin D1 Degradation in Neural Stem Cells Contributes to the Neurogenic Cortical Defects in Down Syndrome
Source: eBioMedicine. 2015 Jan 17;2(2):120–34. doi: 10.1016/j.ebiom.2015.01.010 (PMC4484814; doi:10.1016/j.ebiom.2015.01.010)
Supplement: Supplementary file 1 — Supplementary material. [file mmc1.pdf]

Supplementary Figure 1

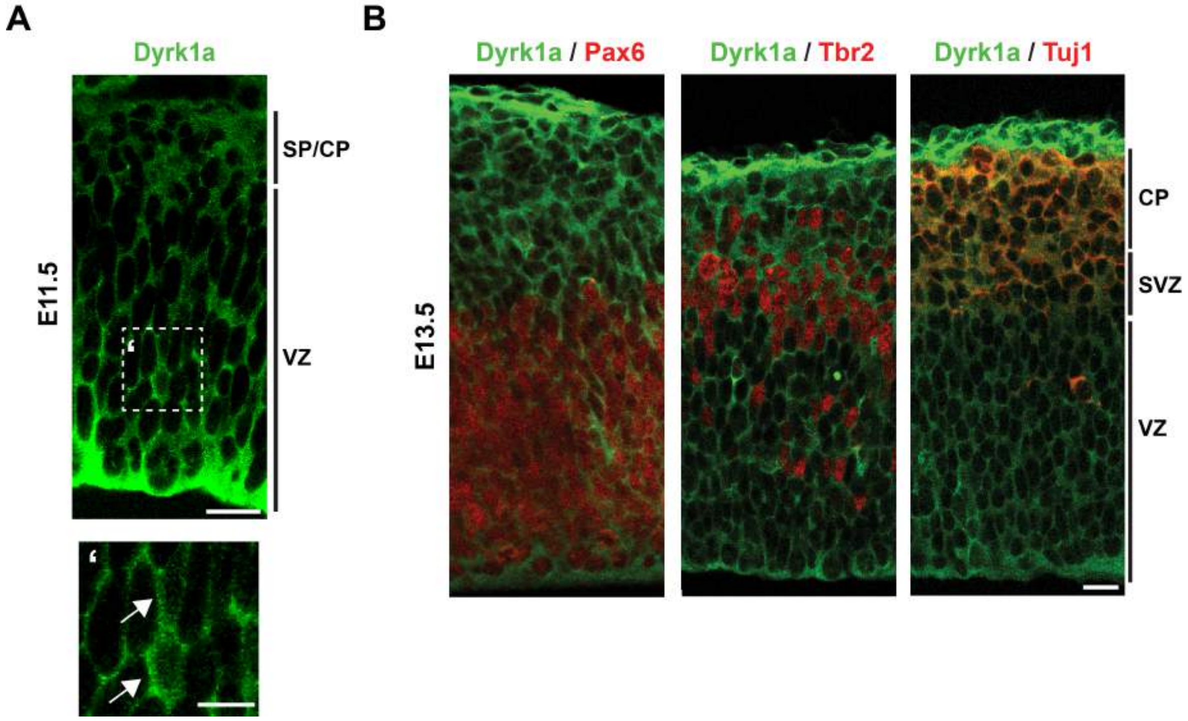

DYRK1A is expressed in progenitors and differentiating neurons of the mouse embryo dorsal telencephalon. (**A**) Confocal images of a coronal E11.5 brain section showing expression of DYRK1A in the cytoplasm of radial glial progenitors (cells in the ventricular germinal layer (VZ)) and in differentiating neurons (cells in the cortical plate (CP)). Arrows in the magnified image (‘) point to progenitors with DYRK1A immunostaining in their nuclei. (**B**) Confocal images of a coronal E13.5 brain section showing DYRK1A immunostaining in radial glial progenitors expressing Pax6 (left), in intermediate progenitors expressing Tbr2 (middle), and in postmitotic cells expressing the neuronal marker Tuj1 (right). SVZ, subventricular germinal zone. Bars = 20 μm (A and B), 10 μm (magnification in A).

Supplementary Figure 2

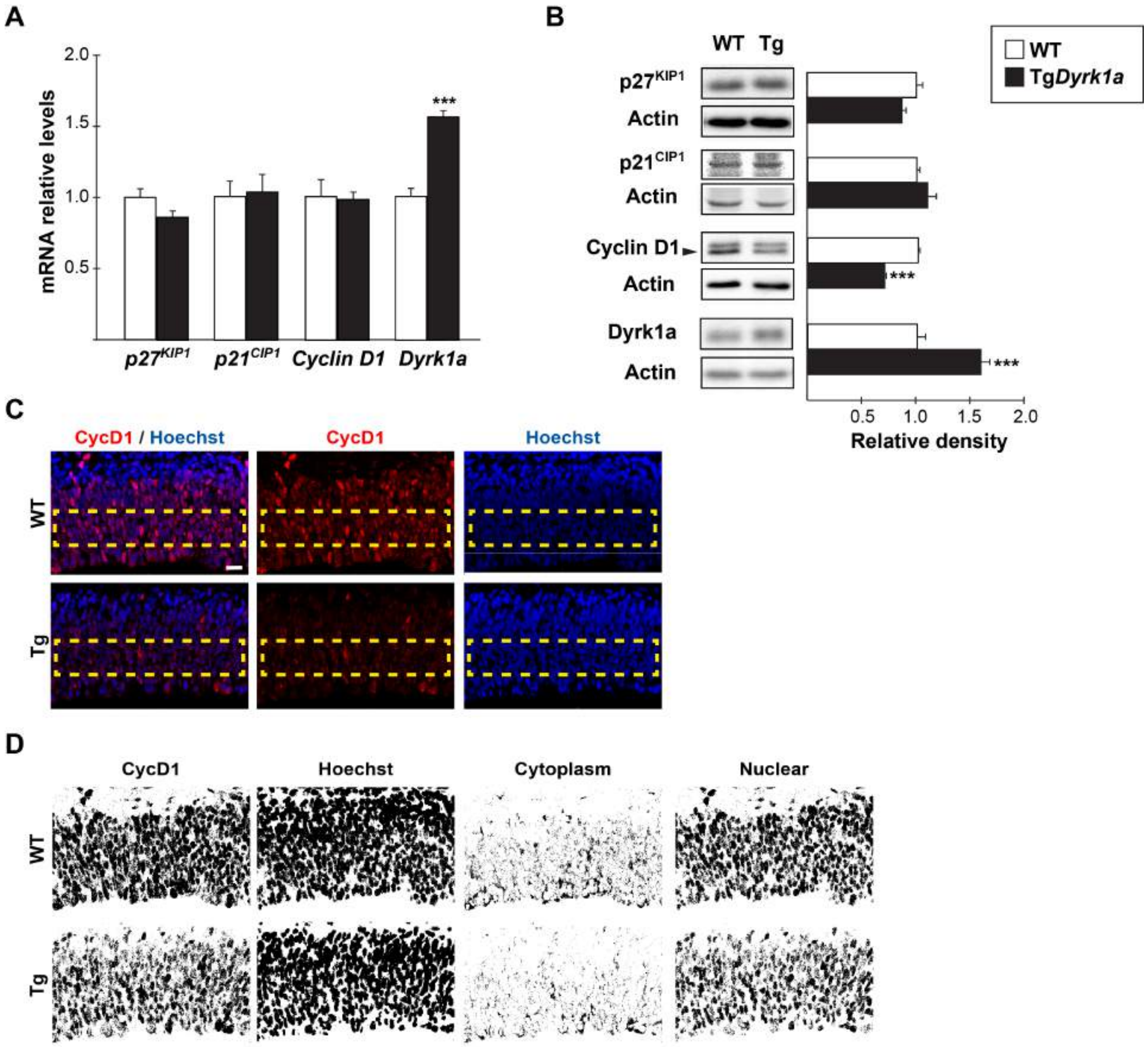

Cyclin D1 protein levels are decreased in Tg*Dyrk1a* embryos. (**A**) Relative mRNA levels of *Dyrk1a*, $p27^{KIP1}$, $p21^{CIP1}$ and *Cyclin D1* determined by RT-PCR on mRNA obtained from wild-type (WT) and Tg*Dyrk1a* embryos. (**B**) Representative Western blots of extracts prepared from WT and Tg*Dyrk1a* embryos and probed with the indicated antibodies, and histograms showing the protein levels in Tg*Dyrk1a* embryos normalized to actin levels and expressed relative to the WTs. Arrowhead indicates the band corresponding to the Cyclin D1 isoform that contains T286. Histogram values are the mean ± S.E.M. ***$P<0.001$ ($n \geq 4$). (**C**) Representative images from E11.5 WT and Tg*Dyrk1a* (Tg) embryos showing Cyclin D1 (CycD1) immunostaining and the nuclei visualized with Hoechst. (**D**) Binary images were generated for quantifications by using the ImageJ software. “Cytoplasm” image was obtained by subtracting the binary “Hoechst” image from the binary “CycD1” image, and “Nuclear” image by subtracting the “Cytoplasm” image from the binary “CycD1” image as indicated in Materials and Methods. Yellow dashed rectangles indicate the region measured. Bar = 20 μm.

Supplementary Figure 3

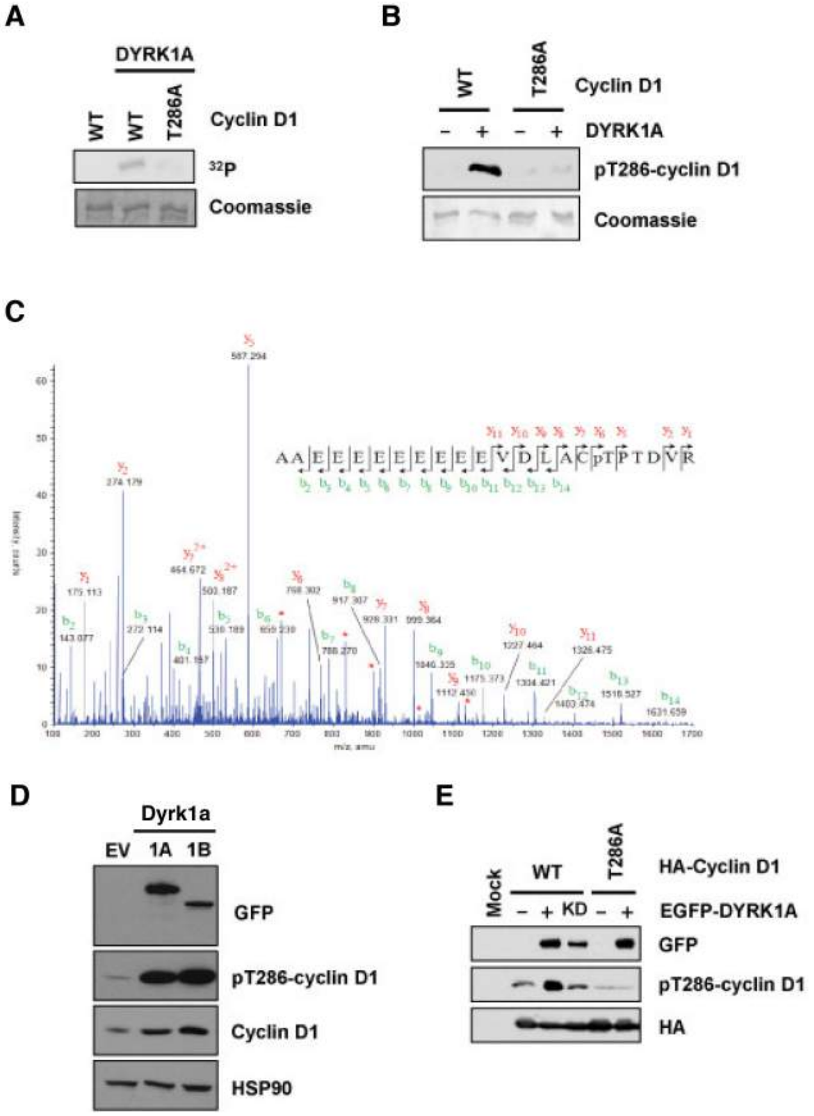

Cyclin D1 is phosphorylated on T286 by DYRK1A. Representative autorad (**A**) and Western blot (**B**) showing phosporylation of Cyclin D1 after the incubation of Wild-type (WT) or T286A GST-Cyclin D1 with recombinant DYRK1A in the presence of [^{32}P]-γ-ATP (in A) or γ-ATP (in B). (**C**) Mass spectrometry analysis of phosphorylated residues on Cyclin D1 after transient co-expression of HA-Cyclin D1 with wild-type EGFP-DYRK1A in HEK293 cells. (**D**) Western blot showing Cyclin D1 phosphorylation on residue T286 after transient co-expression of HA-Cyclin D1 with EGFP-DYRK1A (1A), EGFP-DYRK1B (1B) used as positive control, or empty vector (EV) in HEK293 cells. (**E**) Western blot showing Cyclin D1 phosphorylation on residue T286 after transient co-expression of WT or T286A HA-Cyclin D1 with WT or kinase-dead (KD, K188R) EGFP-DYRK1A in HEK293 cells for 24 h. All data are taken from a single experiment representative of at least two replicate experiments.

Supplementary Figure 4

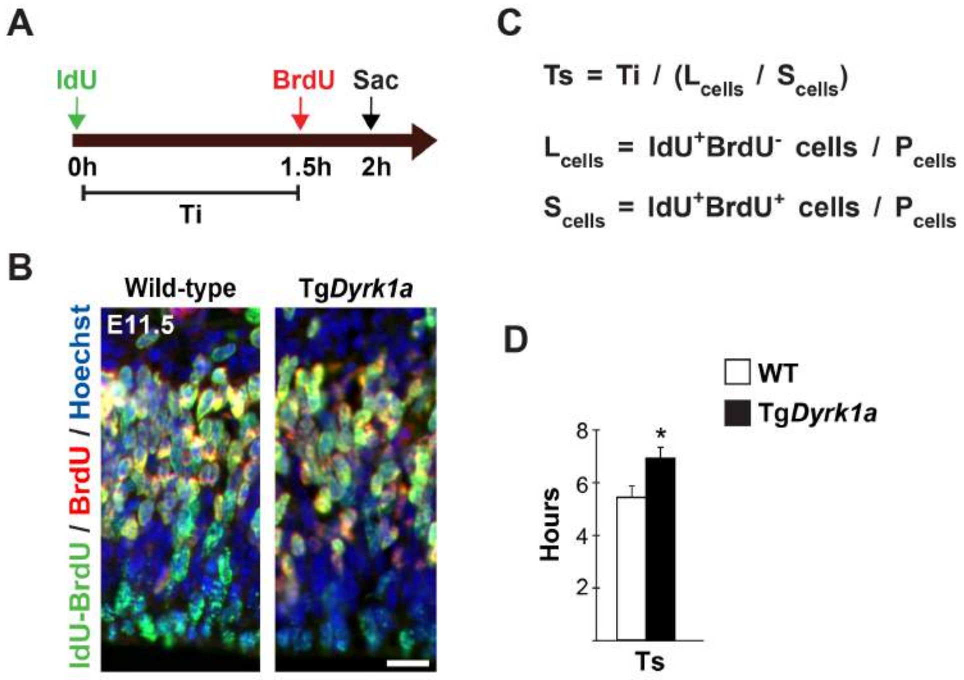

Tg*Dyrk1a* radial glial progenitors have longer S phases than wild-type progenitors. (**A**) Schedule of the double-labelling paradigm used to estimate the S-phase duration (Ts) in E11.5 radial glial progenitors of the dorsal telencephalon. Green and red arrows indicate the time of IdU injection and BrdU injection, respectively. Black arrow indicates the time of sacrifice. (**B**) Representative images of wild-type and Tg*Dyrk1a* brain coronal sections with cells double labelled for IdU and BrdU (green) and only labelled for BrdU (red). Nuclei were visualised with Hoechst. (**C**) Equations showing how Ts was calculated. Ti: time between IdU and BrdU injections; Pcells: number of progenitors; Lcells: proportion of Pcells that have ended the S-phase before the injection with BrdU (green nuclei in B); and Scells: proportion of Pcells that remains in S-phase when BrdU was injected (yellow nuclei in B). (**D**) Histogram showing the estimated Ts value in wild-type and Tg*Dyrk1a* radial glial progenitors. Values are the mean ± S.E.M. *$P<0.05$ (n = 4 per genotype). Bar = 20 μm.

Supplementary Figure 5

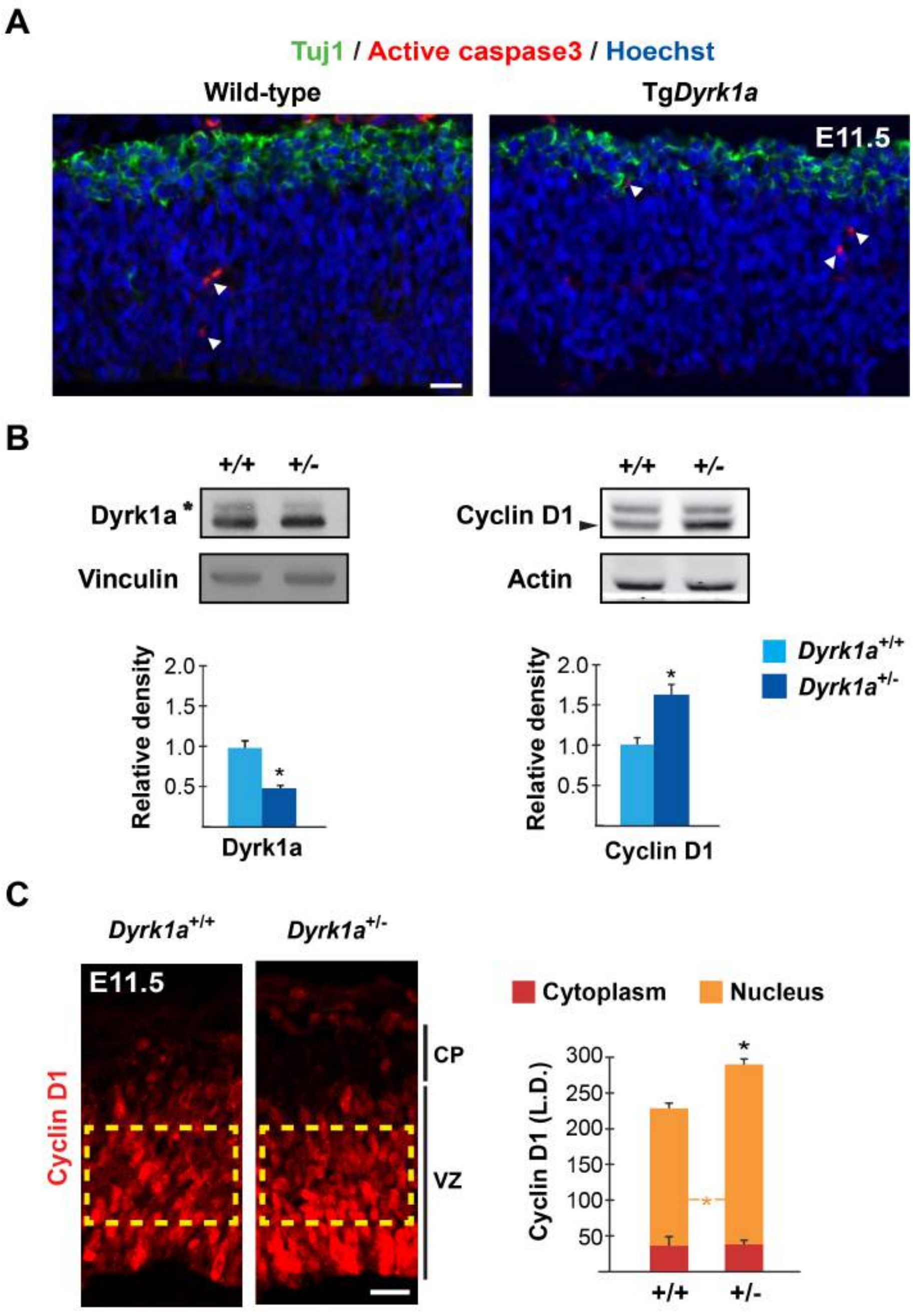

***Dyrk1a* gene-dosage imbalance in radial glial progenitors affects nuclear Cyclin D1 levels but does not affect apoptotic cell death.** (**A**) Representative coronal sections of E11.5 wild-type and Tg*Dyrk1a* embryos immunostained for Tuj1 (green) and active-caspase3 (red) and the nuclei visualized by Hoechst staining (blue). Arrowheads point to cells expressing the active form of caspase3. Cell counts were done in a 400 μm-wide column of the lateral cortical wall. (**B**) Western blots of extracts prepared from the telencephalon of E11.5 *Dyrk1a*$^{+/+}$ (+/+) and *Dyrk1a*$^{+/-}$ (+/-) embryos probed with the indicated antibodies and histograms showing the protein levels of DYRK1A and Cyclin D1 in embryos of the two genotypes normalized to vinculin or actin levels and expressed relative to the WTs. Asterisk indicates the band corresponding to DYRK1A and arrowhead the band corresponding to the

Cyclin D1 isoform that contains T286. (**C**) Coronal brain sections obtained from E11.5 *Dyrk1a*$^{+/+}$ and *Dyrk1a*$^{+/-}$ embryos and immunostained for Cyclin D1. Yellow dashed rectangles indicate the region quantified. Histogram shows the labelling densities (L.D.) of Cyclin D1 fluorescence signals in the nucleus and the cytoplasm of radial glial progenitors (calculated as indicated in Supplementary Figure 2C and 2D). VZ, ventricular zone; CP, cortical plate. Histogram values are the mean ± S.E.M. *$P<0.05$, (n = 4 in B; n = 3 in C). Bar = 20 μm.

Supplementary Figure 6

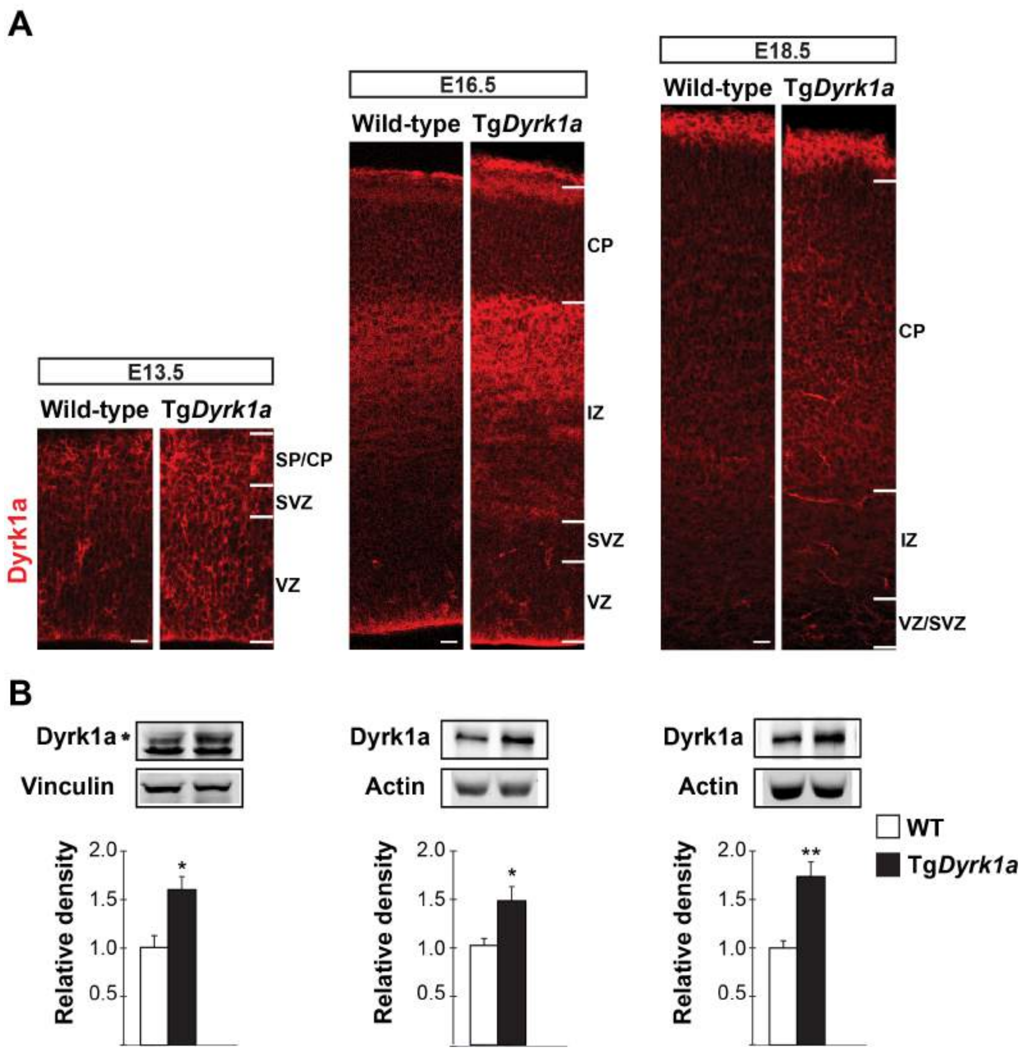

DYRK1A is expressed in wild-type and Tg*Dyrk1a* progenitors and differentiating neurons during the neurogenic phase of cortical development. (**A**) Wild-type (WT) and Tg*Dyrk1a* (Tg) coronal telencephalic sections immunolabelled for DYRK1A at the indicated embryonic stages. CP, cortical plate; IZ, intermediate zone; SP, subplate; VZ, ventricular zone; SVZ, subventricular zone. Bars = 20 μm. (**B**) Western blots of extracts prepared from the dorsal telencephalon of E13.5, E16.5 and E18.5 WT and Tg embryos and probed with the indicated antibodies. Asterisk indicates the band corresponding to DYRK1A at E13.5. Histograms showing the protein levels of DYRK1A normalized to vinculin or actin levels and expressed relative to the WTs. Histogram values are the mean ± S.E.M. *P<0.05, **P<0.01 (n ≥ 3).

Supplementary Figure 7

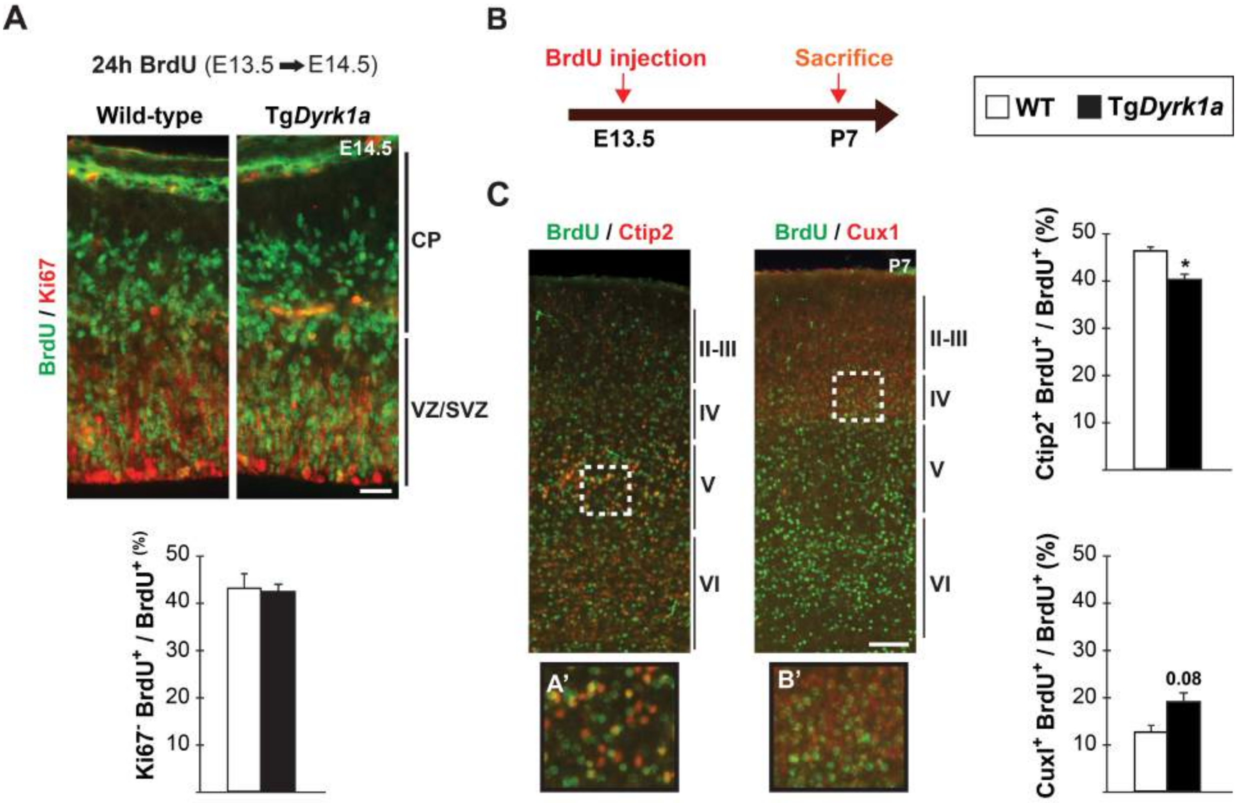

Telencephalic neurons in Tg*Dyrk1a* embryos are produced at normal rates during mid-neurogenesis but they acquire an advanced fate. (**A**) Wild-type (WT) and Tg*Dyrk1a* coronal telencephalic sections immunolabelled for BrdU and Ki67. Pregnant females were injected with BrdU at E13.5 and embryos harvested 24 h later. The histogram shows the percentage of $BrdU^+$ cells that do not express the proliferation marker Ki67. (**B**) Schedule of the cell fate experiment. (**C**) Representative images of the dorsal cortex of P7 WT and Tg*Dyrk1a*. Coronal sections were immunolabelled for BrdU and Ctip2 or for BrdU and Cux1. A' and B' are magnifications of the regions limited by white boxes. BrdU injections were performed at the peak of production of layer V callosal projection neuron (E13.5; Molyneaux et al., 2007). Histograms show the proportion of $BrdU^+$ cells that express the layer V marker Ctip2 or layers II-IV marker Cux1. Note that in Tg*Dyrk1a* embryos there are more $Ctip2^+$ neurons and less $Cux1^+$ neurons, indicating an advanced differentiation program. Cell counts were done in a 100 μm-wide column of the cortical lateral wall. Values are the mean ± S.E.M. *P<0.05 (n = 3). Bars = 20 μm. VZ, ventricular zone; SVZ, subventricular zone; CP, cortical plate.

Supplementary Figure 8

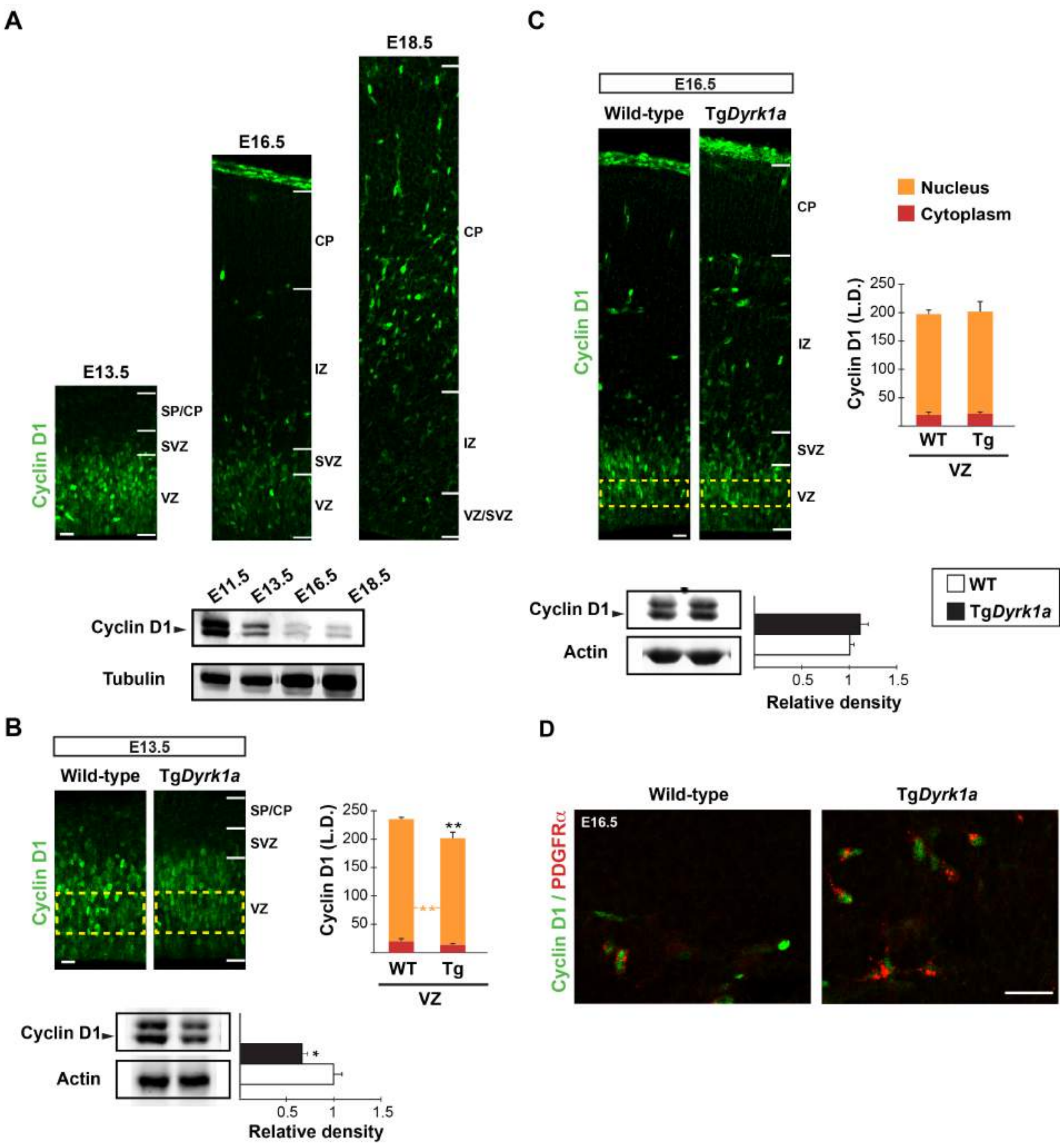

Cyclin D1 expression during corticogenesis in wild-type and Tg*Dyrk1a* embryos. (**A**) Confocal images of wild-type (WT) embryonic brain sections immunolabelled for Cyclin D1 at the indicated developmental stages and Western blot of extracts prepared from the dorsal telencephalon of E13.5, E16.5 and E18.5 WT embryos probed with the indicated antibodies. (**B** and **C**) Representative coronal brain sections obtained from E13.5 (**B**) and E16.5 (**C**) WT and Tg*Dyrk1a* (Tg) embryos and immunostained for Cyclin D1. Yellow dashed rectangles indicate the region quantified. Histograms (on the right) show labelling densities (L.D.) of Cyclin D1 fluorescence signals in the nucleus and cytoplasm of ventricular zone (VZ) radial glial progenitors. L.D. were calculated as indicated in Supplementary Figures 2C and 2D. Western blots of extracts prepared from the dorsal telencephalon of E13.5 (**B**) and E16.5 (**C**)

WT and Tg embryos probed with the indicated antibodies. Arrowhead indicates the band corresponding to the Cyclin D1 isoform that contains T286. Histograms show the protein levels of Cyclin D1 in embryos of the two genotypes normalized to actin levels and expressed relative to the WTs. (**D**) Representative confocal images of E16.5 WT and Tg embryos showing the intermediate zone (IZ) of the dorsal telencephalon immunolabelled for Cyclin D1 and PDGFRα. Histogram values are the mean ± S.E.M. *P<0.05, **P<0.01 (n ≥ 3). CP, cortical plate; SP, subplate; SVZ, subventricular zone. Bars = 20 μm.

Supplementary Figure 9

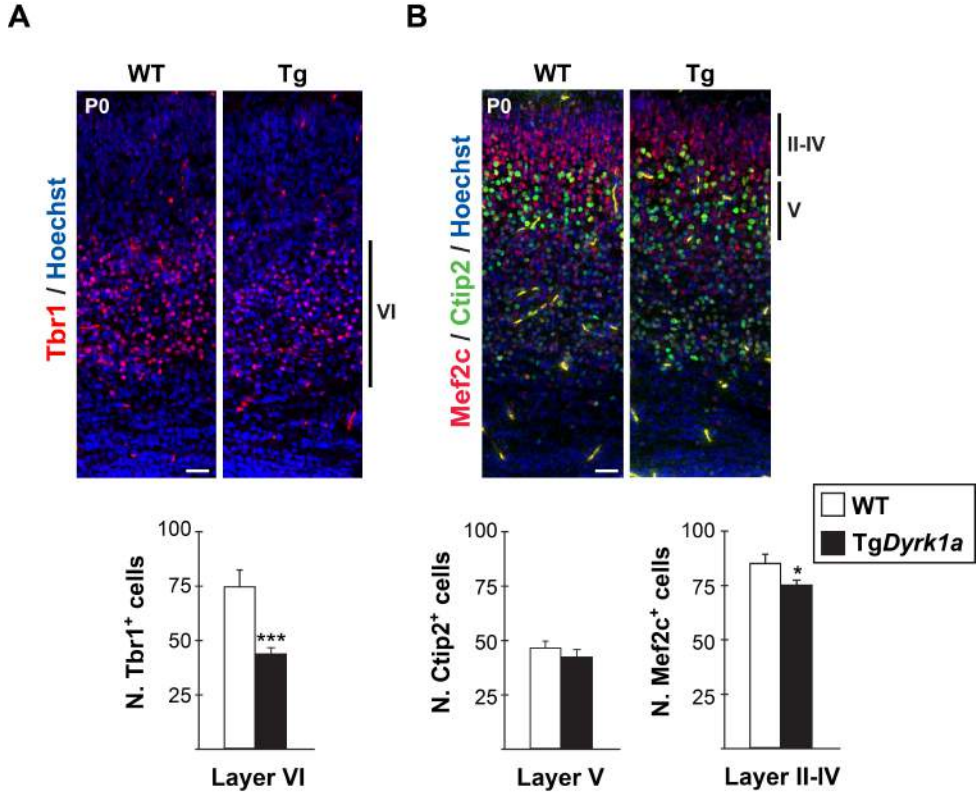

Tg*Dyrk1a* newborn mice show decreased neuronal cellularity in specific cortical layers. Representative coronal sections from P0 wild-type (WT) and Tg*Dyrk1a* (Tg) brains immunostained for Tbr1 (**A**) or Ctip2 and Mef2c (**B**). Histograms show the number of layer VI $Tbr1^{+}$ neurons, layer V $Ctip2^{+}$ neurons and layers II-IV Mef2c neurons in a 100 µm-wide column of the cortical wall. Values are the mean ± S.E.M. *P<0.05, ***P<0.001 (n ≥ 3). Bars = 50 µm.

Supplementary Figure 10

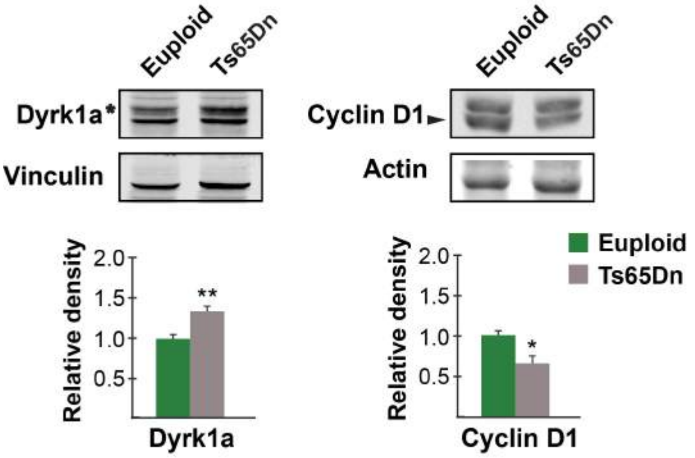

Trisomy of *Dyrk1a* in the Ts65Dn Down syndrome model correlates with a reduction of Cyclin D1 in radial glial progenitors. Representative Western blots of extracts prepared from the telencephalon of E11.5 euploid and trisomic Ts65Dn embryos probed with the indicated antibodies. Histograms showing the protein levels of DYRK1A and Cyclin D1 in Ts65Dn embryos normalized to actin or vinculin levels and expressed relative to the WTs. Asterisk indicates the band corresponding to DYRK1A and arrowhead the band corresponding to the Cyclin D1 isoform that contains T286. *P<0.05, **P<0.01 (n ≥ 3).

Supplementary Figure 11

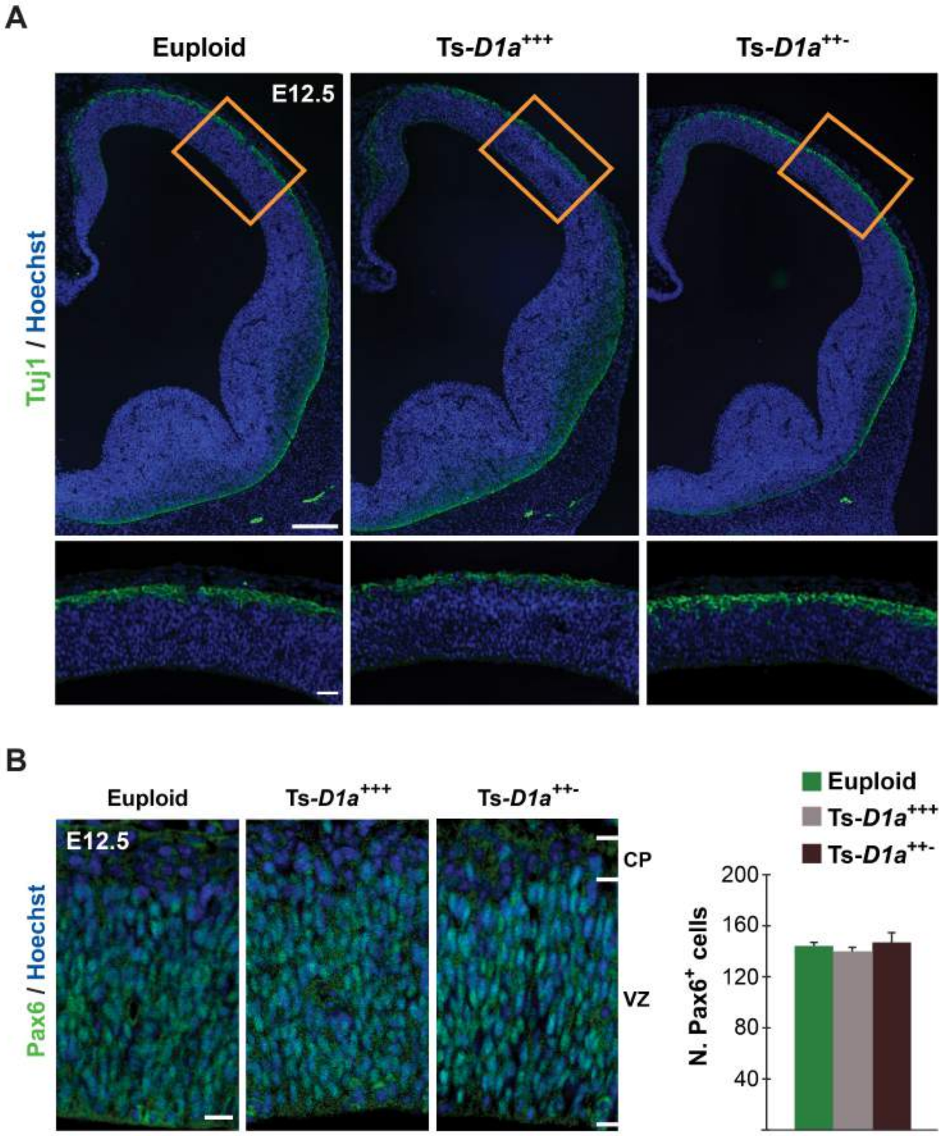

Trisomy of *Dyrk1a* does not affect the number of cortical radial glial progenitors in the Ts65Dn Down syndrome model at the onset of neurogenesis. (**A**) Representative brain sections of euploid, Ts-$D1a^{+++}$ (Ts65Dn) and Ts-$D1a^{++-}$ (Ts65Dn disomic for *Dyrk1a*) E12.5 embryos immunostained for the neuronal marker Tuj1 (green). Nuclei were visualized by Hoechst staining (blue). Yellow squares indicate the regions magnified bellow. Note that immunostaining for Tuj1 is decreased in Ts-$D1a^{+++}$ embryos compared to euploid or Ts-$D1a^{++-}$ embryos (**B**) Representative confocal images from coronal brain sections of E12.5 embryos of the indicated genotypes immunostained for Pax6. Histogram shows the total number of $Pax6^{+}$ radial glial progenitors in a 100 μm-wide column of the lateral cortical wall. CP, cortical plate; VZ, ventricular zone. Histogram values are the mean ± S.E.M. *P<0.05, **P<0.01 (n ≥ 3). Bars = 200 μm in A and 20 μm in B.

Supplementary Figure 12

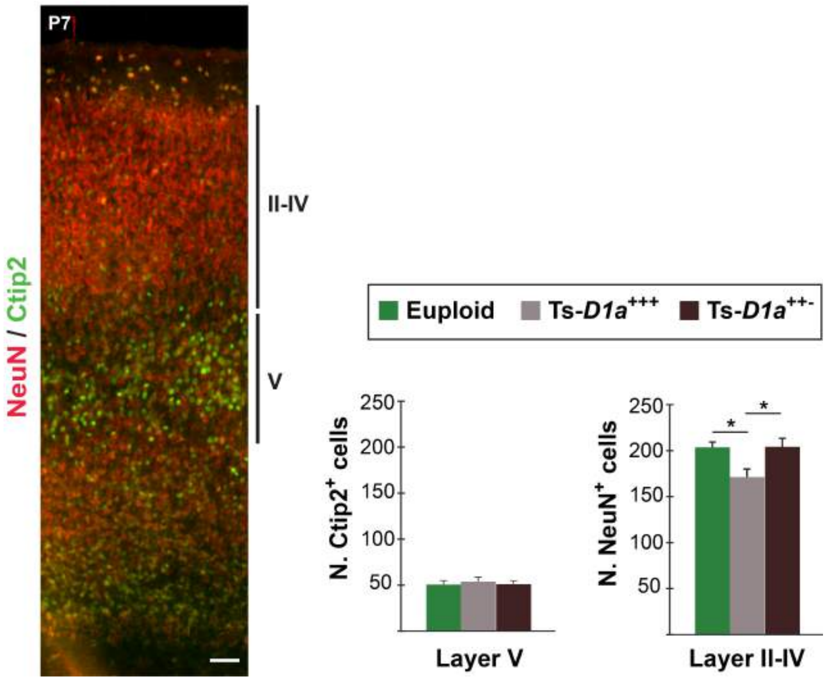

Trisomy of *Dyrk1a* alters the number of upper layers cortical neurons in the Ts65Dn Down syndrome model. Representative image from a coronal brain section of P7 mice immunolabelled for Ctip2 (green) and NeuN (red). Histograms show the number of layer V Ctip2^{+} neurons and layer II-IV NeuN^{+} neurons in a 100 μm-wide column of the cortical wall of the indicated genotypes. Histogram values are the mean ± S.E.M. *P<0.05 (n = 4). Bars = 50 μm.

Supplementary Figure 13

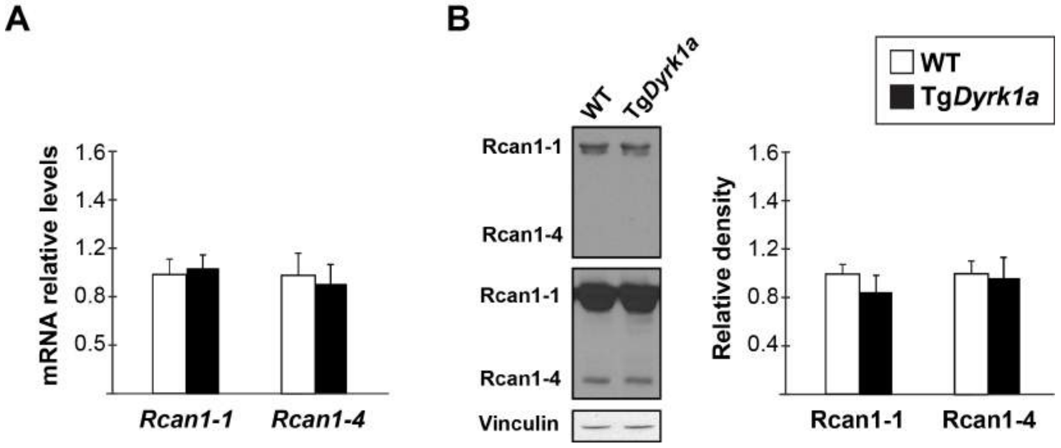

Triplication of *Dyrk1a* in radial glial progenitors does not affect Rcan1 levels. (**A**) Relative expression of *Rcan1-1* and *Rcan1-4* transcripts determined by RT-PCR on mRNA obtained from the telencephalon of E11.5 wild-type (WT) and Tg*Dyrk1a* embryos. (**B**) Representative Western blot of extracts prepared from the telencephalon of E11.5 embryos and probed with an antibody that hybridizes with Rcan1-1 and Rcan1-4 isoforms (Porta et al., 2007). Two different exposure times are shown. Histograms show the protein levels in Tg*Dyrk1a* embryos normalized to vinculin and expressed relative to the WTs. Histogram values are the mean ± S.E.M. (n ≥ 3). Differences between genotypes were not statistically significant.

SUPPLEMENTARY MATERIALS AND METHODS

DNA plasmids

pcDNA3-HA-cyclin D1 plasmids were reported previously (Densham et al., 2008) whilst pEGFPC3-DYRK1A, wild-type and kinase-dead (K188R), was kindly provided by Walter Becker (Institut für Pharmakologie und Toxikologie, Aachen, Germany).

In vitro DYRK kinase assay

0.02 µg recombinant DYRK1A (Millipore) was assayed for Cyclin D1 kinase activity by incubating with recombinant GST-Cyclin D1 beads in the presence of 50 mM Tris/HCl, pH 7.5, 0.1 mM EGTA, 0.1% (v/v) 2-mercaptoethanol, 10 mM MgCl2, 0.1 mM [γ-^{32}P]-ATP in a total volume of 50 µl for 30 min at 30°C as describe previously (Lochhead et al., 2005). Terminated reactions were separated by SDS-PAGE and transferred onto PVDF membrane and analysed by autoradiography or by immunoblotting.

Analysis of Cyclin D1 phosphorylation by mass spectrometry

HA-Cyclin D1 was expressed with wild-type EGFP-DYRK1A in HEK293 cells for 24 h. Cyclin D1 was isolated from whole cell lysate by immunoprecipitation with anti-HA antibody conjugated to agarose beads (Santa Cruz). The immune-complexes were fractionated by SDS-PAGE and the resulting gel was stained with Simply Blue Safe Stain (Invitrogen). The Cyclin D1 band was excised, digested with trypsin and analysed by mass spectrometry essentially as described previously (Ashford et al., 2014), except that a Qstar Pulsar i mass spectrometer (Applied Biosystems/MDS Sciex) was used. For targeted LC-MS/MS analyses, product ion spectra of the triply charged ions corresponding to the non-, mono-, and di-phosphorylated Cyclin D1 peptide Ala268-Arg291 were recorded continuously.

Estimation of the S-phase duration by the double-labelling paradigm

Estimation of S-phase duration (Ts) was performed following the double-labelling protocol described in Martynoga et al. (2005). Briefly, a E11.5 pregnant female was intraperitoneally injected with 5-Iodo-2′-deoxyuridine (IdU, Sigma; 50 µg per gram body weight), and 1.5 h later with 5-bromo-2′-deoxyuridine (BrdU, Sigma; 50 µg per gram body weight). Sacrifice was 30 min after the last injection (see schedule in Supplementary Figure 4A). Embryo brains were processed and cryosectioned and sections were immunolabelled using an antibody that only recognizes BrdU and an antibody that recognizes both, IdU and BrdU. Radial glial progenitors that were labelled only with IdU or colabeled with IdU-BrdU were counted in a 250 µm-wide field of the dorsolateral wall in a minimum of 3 sections per embryo. Calculation of the Ts (S-phase duration) was performed following the equation shown in Supplementary Figure 4C, in which Ti is the time between IdU and BrdU injections; Pcells is the number of progenitors; Lcells is the proportion of Pcells that have ended the S-phase before the injection with BrdU and, therefore, are only labelled with IdU; and Scells is the proportion of Pcells that remains in S-phase when BrdU was injected, cells colabelled with IdU and BrdU.

Primary antibodies used for immunostainings

Antibody	Host	Source	Reference	Dilution
Active caspase3*	Rabbit	BD Bioscience	559565	1:500
BrdU	Rat	Abd Serotec	OBT0030G	1:500
BrdU	Mouse	Hybrydoma Bank	G3G4	1:50
BrdU-IdU	Mouse	Becton Dickinson	347580	1:50
Cyclin D1	Rabbit	Thermo Scientifics	RM-9104	1:100
Ctip2*	Rat	Abcam	ab18465	1:250
CuxI*	Goat	Santa Cruz Biotechnology	sc-6327	1:50
DYRK1A	Mouse	Abnova	H00001859	1:250
Ki67*	Rabbit	Abcam	ab15580	1:100
Mef2c*	Rabbit	Abcam	ab64644	1:100
NeuN	Mouse	Millipore	MAB377	1:500
Olig2	Rabbit	Millipore	AB9610	1:500
Pax6*	Mouse	Hybrydoma Bank	Pax6	1:50
Pax6	Rabbit	Covance	PRB-278P	1:500
PDGFRα	Rat	BD Pharmigen	558774	1:100
pH3	Rat	Sigma-Aldrich	H 9908	1:200
Tbr1*	Rabbit	Abcam	ab31940	1:200
Tbr2* #	Rabbit	Abcam	ab23345	1:200
TujI	Mouse	Covance	#MMS435P	1:500
TujI	Rabbit	Sigma-Aldrich	T2200	1:500

* Citrate pretreatment
# For EdU labelling this antibody was used without antigen retrieval

Oligonucleotide primers used for Real Time qPCR

Gene		Primer sequence 5'-3'	Size (bp)
CyclinD1	F	CACAACGCACTTTCTTTCCA	88
	R	TGACTCCAGAAGGGCTTCAA	
Delta1	F	CGGCTCTTCCCCTTGTTCTAA	126
	R	GGGGAGGAGGCACAGTCATC	
Dyrk1a	F	ATCCAGCAACTGCTCCTCTG	140
	R	CCGCTCCTTCTTATGACTGG	
$p21^{CIP}$	F	TTGTCGCTGTCTTGCACTCT	103
	R	AATCTGTCAGGCTGGTCTGC	
$p27^{KIP1}$	F	TTGGGTCTCAGGCAAACTCT	131
	R	TCTGTTCTGTTGGCCCTTTT	
Ppia	F	ATGGCAAGACCAGCAAGAAG	143
	R	TTACAGGACATTGCGAGCAG	
Rcan 1-1	F	CCGTAGGGTGACTCTG	243
	R	GCTCTTAAAATACTGGAAGGT	
Rcan 1-4	F	GCGAGTCGTTCGTTAAG	185
	R	ATACTGGAAGGTGGTGT	

SUPPLEMENTARY REFERENCES

Densham, R.M., Todd, D.E., Balmanno, K., and Cook, S.J. (2008). ERK1/2 and p38 cooperate to delay progression through G1 by promoting cyclin D1 protein turnover. Cell Signal *20*, 1986-1994.

Lochhead, P.A., Sibbet, G., Morrice, N., and Cleghon, V. (2005). Activation-loop autophosphorylation is mediated by a novel transitional intermediate form of DYRKs. Cell *121*, 925-936.

Porta, S., Marti, E., de la Luna, S., and Arbones, M.L. (2007). Differential expression of members of the RCAN family of calcineurin regulators suggests selective functions for these proteins in the brain. Eur J Neurosci *26*, 1213-1226.
